# Internal Polarization Field Induced Hydroxyl Spillover Effect for Industrial Water Splitting Electrolyzers

**DOI:** 10.1007/s40820-023-01253-9

**Published:** 2023-11-30

**Authors:** Jingyi Xie, Fuli Wang, Yanan Zhou, Yiwen Dong, Yongming Chai, Bin Dong

**Affiliations:** State Key Laboratory of Heavy Oil Processing, College of Chemistry and Chemical Engineering, China University of Petroleum (East China), Qingdao, 266580 People’s Republic of China

**Keywords:** Hydroxyl spillover effect, Internal polarization field, Heterostructure, Oxygen reduction reaction, Anion exchange membrane water electrolysis

## Abstract

The formation of multiple oxygen intermediates supporting efficient oxygen evolution reaction (OER) are affinitive with hydroxyl adsorption. However, ability of the catalyst to capture hydroxyl and maintain the continuous supply at active sits remains a tremendous challenge. Herein, an affordable Ni_2_P/FeP_2_ heterostructure is presented to form the internal polarization field (IPF), arising hydroxyl spillover (HOSo) during OER. Facilitated by IPF, the oriented HOSo from FeP_2_ to Ni_2_P can activate the Ni site with a new hydroxyl transmission channel and build the optimized reaction path of oxygen intermediates for lower adsorption energy, boosting the OER activity (242 mV vs. RHE at 100 mA cm^–2^) for least 100 h. More interestingly, for the anion exchange membrane water electrolyzer (AEMWE) with low concentration electrolyte, the advantage of HOSo effect is significantly amplified, delivering 1 A cm^–2^ at a low cell voltage of 1.88 V with excellent stability for over 50 h.

**Supplementary Information:**

The online version contains supplementary material available at 10.1007/s40820-023-01253-9.

## Highlights

- Analyzed the function of internal polarization field in Ni_2_P/FeP_2_ via hydroxyl spillover effect.
- From theoretical design to experimental verification, to optimize adsorption energy of oxygen intermediates on Ni active site, and further boost the xygen evolution reaction process.
- A hydroxyl spillover effect driven by internal polarization field in Ni_2_P/FeP_2_ can be amplified in low concentration alkaline electrolyte environment, and facilitate the application in anion exchange membrane water electrolyzer systems.

**Supplementary Information:**

The online version contains supplementary material available at 10.1007/s40820-023-01253-9.

## Graphical abstract

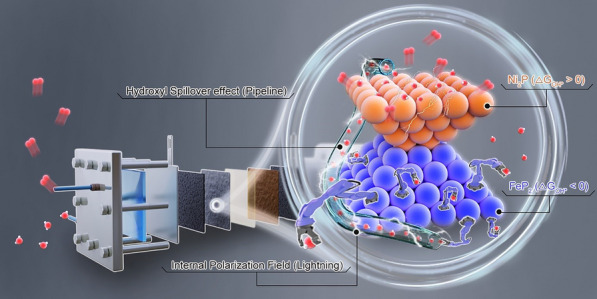

## Introduction

Due to the compact design and fast system response under widespread current density operation, proton exchange membrane water electrolyzer (PEMWE) received unprecedented attention for high purity hydrogen production, especially with intermittent hybrid wind-solar integrated energy system [1–3]. However, the harsh acidic environment forces the selection of precious metal-based catalysts as electrodes, which prevented commercial PEMWE from large-scale practical application [4]. On the contrast, transition metal (TM)-based catalysts with cost advantages and commercial prospects are favorable to show reasonable activity and stability in alkaline media [5–7]. In fact, two kinds of electrolyzer technologies using basic liquid electrolyte are alkaline water electrolyzer (AWE) and anion exchange membrane water electrolyzer (AEMWE) [8–10]. In addition to long start-up preparation and slow response to changes in electric power load, the development of AWE for further practical applications is dramatically hindered by the sluggish kinetics of oxygen evolution reaction (OER) with four-concerted proton-electron transfer (CPET) pathways [11–13].

**Fig. 1:**
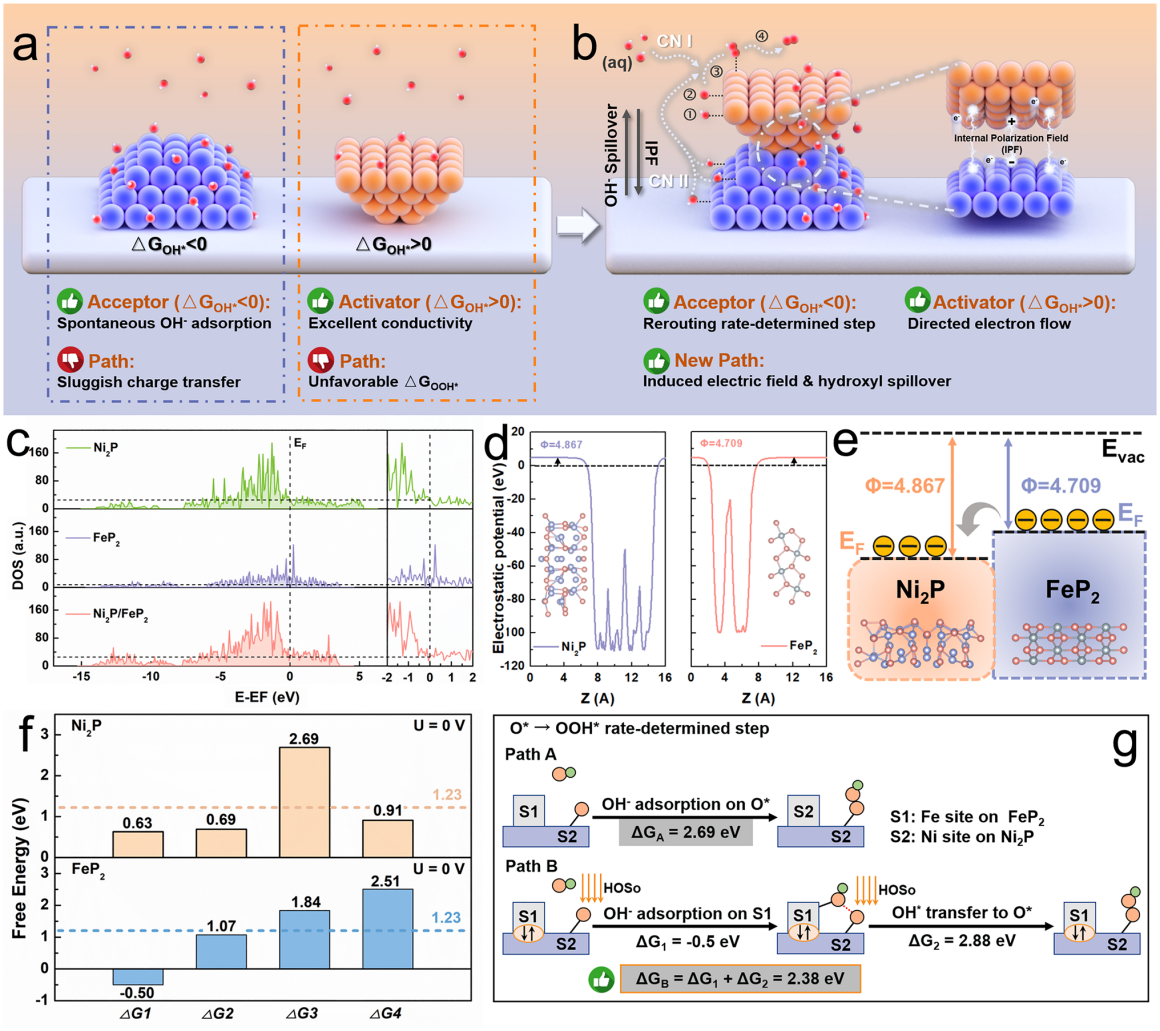
**a, b** Rationales of material construction for hydroxyl spillover in water electrolysis. herein the serial numbers indicate: 1, hydroxyl adsorbates’ evolution; 2, Deprotonation; 3, Oxygen intermediate evolution; and 4, O_2_ release.** c **DOSs of Ni_2_P/FeP_2_, FeP_2_, and Ni_2_P. **d** The computed work functions of Ni_2_P and FeP_2_ in Ni_2_P/FeP_2_/MN. **e** Schematic energy band diagrams of the Ni_2_P/FeP_2_/MN heterostructure and the Fermi level (EF), work function (*Φ*), vacuum level (*E*_vac_). **f** Gibbs free energies of Ni_2_P and FeP_2_ for the four-step OER process. **g** Gibbs free energy of the O* → HO* rate-determined step of of Ni_2_P/FeP_2_ with different reaction paths

From Sabatier’s principle, an ideal OER catalyst requires a moderate adsorption strength with oxygen intermediates, that is, the interaction should be neither too strong nor too weak [14]. As one of the well-acknowledged mechanisms of OER, adsorbate evolution mechanism (AEM) with metal bands serving as the redox center proceeds via multiple oxygen intermediates (OH*, O*, OOH*, and O_2_) [15]. In typical CPET process, the existence of OH^−^ mainly determines the Gibbs free energies (Δ*G*) of two steps: i) generation of OH* radical (Δ*G*_OH*_, * + OH^−^ → OH* + e^−^) via adsorption of OH^−^ at an active site (*), which is the primary step of CPET process; ii) generation of the intermediate OOH* (Δ*G*_OOH*_, *O + OH^−^ → OOH* + e^−^) via the nucleophilic attack of OH^−^ on O*, which is generally considered to be the rate-determining step (RDS) with high energy barrier, especially in the condition of traditional AWE system operated in concentrated KOH (typically 30 wt%) electrolytes [16]. On these accounts, the regulation of concentration and adsorption effect of OH^−^ are the fundamental way to navigate the RDS and optimize OER catalytic activity [17, 18]. As shown in Fig. 1a, there are two cases of Δ*G*_OH*_ in current research, one is that an acceptor can well perform in spontaneous OH^−^ adsorption (Δ*G*_OH*_ < 0); the other one is that an activator with good conductivity and fast charge transfer, but the adsorption energy barrier of OH* at the active site is high (Δ*G*_OH*_ > 0), which is hard to capture OH^−^. However, due to the too positive or negative Δ*G*_OH*_, many transition metal (TM)-based catalysts exhibit poor OER kinetics, limiting the sustainable development of hydrogen economy [19, 20].

By comparison, AEMWE as an emerging technology has combined the advantages of both AWE (cost-effective and naturally abundant materials) and PEMWE (fast response, mild reaction conditions, membrane separation, and high current density) [21, 22]. Nevertheless, the regulation of OH^−^ concentration at the active site becomes more important, for the reason that most common electrolyte in AEMWE is pure water or low concentrated KOH solution [23, 24]. Compared with AWE system, low concentrated electrolyte results in insufficient supply of OH^−^. This phenomenon is more obvious in the two CPET steps mentioned above, which greatly limits the overall efficiency of hydrogen production in AEMWE [25, 26]. Hence, one of the biggest challenges of AEMWE is to design a high-efficiency and low-cost OER catalyst, with enhanced OH^−^ capture ability or newly OH^−^ supply transmission channel, to accelerate the mass transfer process and achieve industrial efficient hydrogen production.

Herein, we presented a non-precious Ni_2_P/FeP_2_ electrocatalytic platform with heterostructure by introducing the hydroxyl spillover (HOSo) effect, in which FeP_2_ and Ni_2_P functionalize as hydroxyl acceptor and activator (Fig. 1b), respectively. Benefiting from the spontaneous OH^−^ adsorption of FeP_2_ and the internal polarization field (IPF) in the heterojunction system, the supply of hydroxyl can be driven by a new path. This can further reduce the RDS energy barrier of Ni_2_P/FeP_2_ and optimized the electronic structure of Ni active site. The well-designed catalyst exhibited an overpotential (*ƞ*_100_, 100 mA cm^–2^) of only 242 mV for AWE system, and showed high performance (1 A cm^–2^ at 1.88 V) in AEMWE system as well. It is encouraging to note that due to the additional hydroxyl supply path, the potential differences (Δ*E*) between heterostructure and Ni_2_P/MN in 0.1 M KOH and 1.0 M phosphate buffer solution (PBS (pH = 7)) are 1.6 and 3.4 times of that in 1.0 M KOH at 50 mA cm^–2^ in AWE, and even 1.9 and 5 times in AEMWE, respectively. Therefore, for favorable OER process, compared to the OH^−^ provided from electrolyte (Channel I, CN I), the extra OH^−^ captured by FeP_2_ and transferred to Ni active site in Ni_2_P (Channel II, CN II) is more crucial when OH^−^ concentration is low. The interesting hydroxyl dual-channel may be responsible for high catalytic activity. This research proposed a new strategy through the incorporation of HOSo effect, which could construct new reaction path and enhance the OER catalytic performance in low concentration alkaline electrolyte and even pure water in both AWE and AEMWE system.

## Results and Discussion

### Theoretical Viewpoint for Hydroxyl Dual-Channel Transmission Process

The rationality of configuration for catalyst with IPF induced hydroxyl dual-channel transmission is predicted by density functional theory (DFT) calculations. Figure S1 shows the Ni_2_P/FeP_2_ heterostructure model, and the atomic models of Ni_2_P and FeP_2_ are presented in Figs. S2 and S3. Their electronic structures were further investigated by the density of states (DOS) [27]. As compared with FeP_2_, larger occupation near the Fermi level (*E*_*F*_) is observed for Ni_2_P and Ni_2_P/FeP_2_ (Fig. 1c), indicating the heterostructure inherits the excellent conductivity of Ni_2_P, which is conducive to the electron transport process in water electrolysis [28]. As illustrated in Fig. 1d, the work function of Ni_2_P (4.867 eV) is obviously higher than FeP_2_ phase (4.709 eV), suggesting the possible homogenization of multiple intermediates’ adsorption energy due to the strong electron interaction at the heterointerface [29]. Consequently, the potential differences will be generated at interface domain and the electrons will transfer from FeP_2_ to Ni_2_P spontaneously inside the heterostructure (Fig. 1e), which leads to the formation of IPF pointing from positively charged Ni_2_P to the negatively charged FeP_2_ region [30–32]. As a more intuitive evidence, planar average potential along the Z-direction of Ni_2_P/FeP_2_ system was calculated (Fig. S4). The electrostatic potential energy level of Ni_2_P is much lower than that of FeP_2_, corresponding to a higher work function. This is consistent with the results of the work function calculation in Fig. 1d. Thus, there exist IPF in the Ni_2_P/FeP_2_ system, in which electrons can spontaneously transfer from FeP_2_ to Ni_2_P. Such an IPF would provide extra hydroxyl supply channel via driving HOSo from FeP_2_ to Ni_2_P, boosting the OER. The work functions difference (Δ*Φ*) between two materials was 0.158 eV, and the IPF potential (Δ*U*) (= Δ*Φ*/e, e is the electron charge) was calculated by *E* = Δ*U*/*d*, in which the thickness of stacking layer (*d*) is 10 Å [33]. Thus, the IPF strength was roughly estimated to be 1.58 × 10^8^ V m^−1^.

To reveal the origin of adsorption properties optimization, the projected density of states (PDOS) of Ni_2_P, FeP_2_ and Ni_2_P/FeP_2_ models (Fig. S5) were analyzed [34]. It was found that the d band center (*ε*_*d*_) of Ni sites for Ni_2_P is at −1.85 eV whereas the *ε*_*d*_ for heterostructure is downward shifted to −2.09 eV. Simultaneously, the *ε*_*d*_ of Fe sites for FeP_2_ (−0.87 eV) is also downward shifted after forming the heterojunction (−1.08 eV). According to the d-band theory, a downward shift of the d states of Ni and Fe sites with respect to the Fermi level results in reduced occupancy of antibonding states with adsorbed oxygen intermediates, implying the optimal binding strength of the oxygen species and optimized Gibbs free energy [35].

The interface effect on OER kinetics was further investigated via well-established CPET pathway with Ni active site (act_Ni_, Fig. S6) and Fe active site (act_Fe_, Fig. S7), respectively [36]. The RDS of Ni_2_P/FeP_2_ with act_Ni_ possesses the lowest energy barrier of 2.38 eV (Fig. S8), compared with Ni_2_P (2.69 eV) and FeP_2_ (2.51 eV) in Fig. 1f, as well as the Ni_2_P/FeP_2_ with act_Fe_ (2.87 eV, Fig. S9). Thus, the OER kinetics of Ni_2_P/FeP_2_ would overcome the electron-transfer limitation and become hydroxyl-transfer-determining [25]. Correspondingly, the possible mechanism of the optimized Gibbs free energy was demonstrated in Fig. 1g. The non-spontaneous adsorption of hydroxyl (Δ*G*_OH*_ > 0) leads to higher energy consumption of pure Ni_2_P (0.63 eV) in OER process. Moreover, in the subsequent step, O* at Ni site would also be more difficult to bind to the OH^−^ in electrolyte directly, resulting in a higher energy barrier (2.69 eV) for the formation of OOH* in RDS, which is unfavorable to the oxygen evolution process (Path A) [37]. By the contrast, cooperating with the ability of FeP_2_ (−0.5 eV) to absorb spontaneously (Δ*G*_OH*_ < 0) and IPF at the heterojunction interface, hydroxyl captured at Fe site would migrate to the Ni site and combined with O* to form OOH* intermediate (Path B), in which the energy barrier (2.38 eV) is lower than Path A. Therefore, the hydroxyl supply at Ni site in Ni_2_P/FeP_2_ heterostructure comes from dual channel: one is from the electrolyte (CN I), and the other is from the OH^−^ captured by FeP_2_ overflow under the function of IPF (CN II). The new pathway driven by IPF will provide extra hydroxyl supply for OOH* formation at the Ni active site, thereby reducing the energy barrier of the resolution step. The interfacial hydroxyl spillover routes of Ni_2_P/FeP_2_ were simulated to elucidate how HOSo effect contributes to the overall OER activity and how IPF affects the kinetics of the interfacial HOSo. Accordingly, Fig. S10 is the HOSo routes of Ni_2_P (Fig. 10a) and Ni_2_P/FeP_2_ (Fig. 10b). Notably, the Δ*G*_OH(TS)_ at the heterojunction interface of Ni_2_P/FeP_2_ (0.96 eV) is much lower than that of Ni_2_P (1.82 eV) in Fig. S11. Thus, the migration of OH to Ni_act_ on heterojunction interface of Ni_2_P/FeP_2_ is easier, which can facilitate the continuation of OER process.

In addition, the adsorption energy of H_2_O on the catalyst surface is also regarded as an important index to evaluate the performance of OER [38]. As shown in Fig. S12, the water adsorption energy on FeP_2_ is higher than Ni_2_P, indicating that FeP_2_ could adsorb H_2_O easier. In a nutshell, DFT calculations certify that coupling FeP_2_ with Ni_2_P could drive the supply of hydroxyl by dual channel under the IPF and optimize the adsorption of OER intermediates, which can empower Ni_2_P/FeP_2_ to apply to high/low OH^−^ concentration electrolyte conditions in both AWE and AEMWE system.

### Material Synthesis and Characterization

**Fig. 2:**
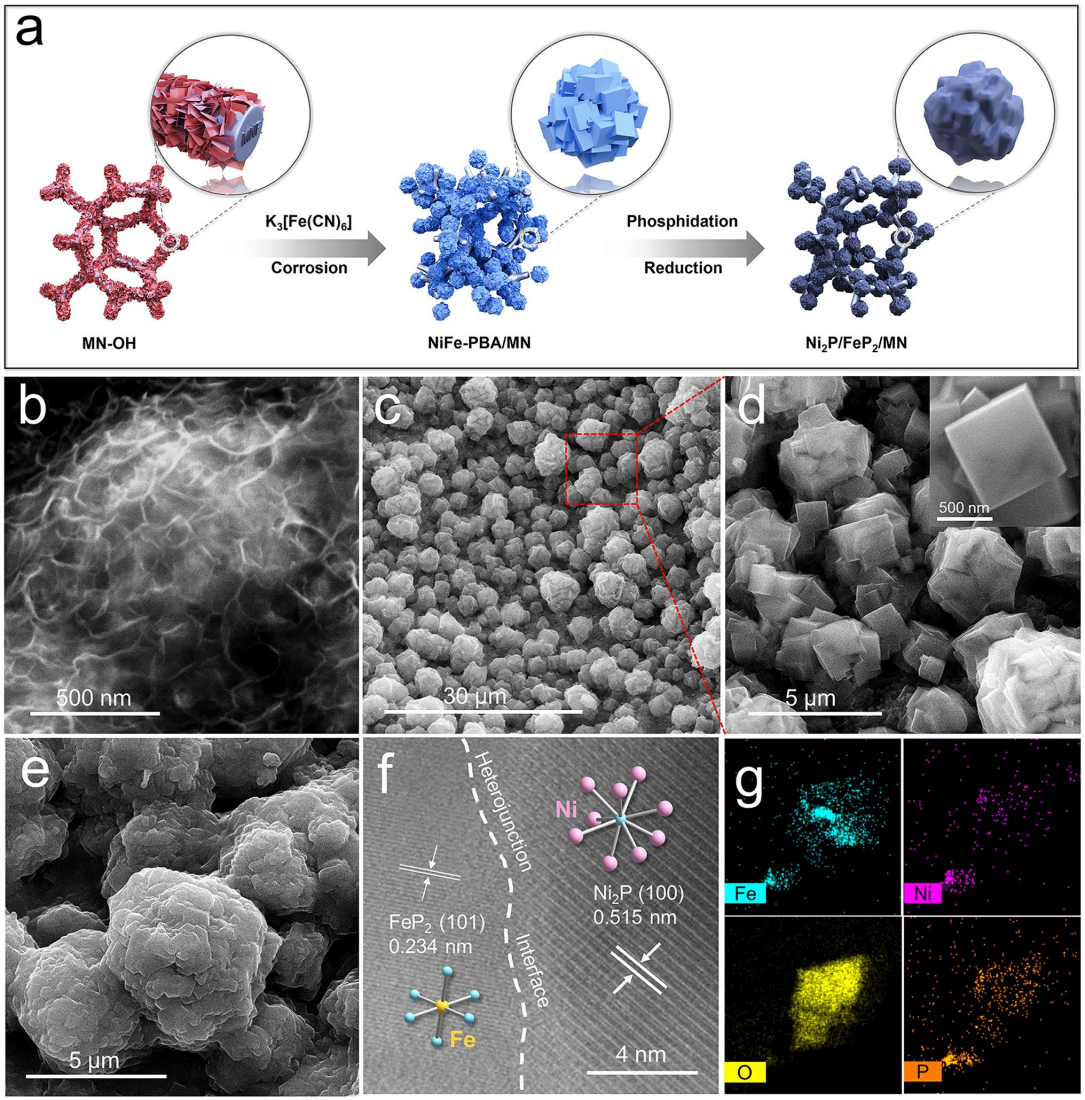
**a** Schematic illustration of the formation process of Ni_2_P/FeP_2_/MN, SEM images of **b** MN–OH, **c, d** NiFe-PBA/MN, **e** Ni_2_P/FeP_2_/MN; HRTEM image **f** and TEM mapping **g** of Ni_2_P/FeP_2_/MN

To test the hypothesis, Ni_2_P was intimately combined with FeP_2_ by hydrothermal and phosphating processes. The synthesis steps of Ni_2_P/FeP_2_/MN heterostructures are schemed in Fig. 2a, and the fabrication details are available in experimental procedures. As shown in Figs. 2b and S13, the scanning electron microscopy (SEM) image of MN-OH demonstrated interconnected nanosheets that uniformly distributed on the molybdenum nickel (MN) skeleton, and the MN can provide enough Ni source, high mechanical strength and good electrical conductivity. After redox reaction with K_3_[Fe(CN)_6_], a spherical Rubik’s cube framework (Figs. 2c and S14a–c) was constructed by stacking in-situ grown NiFe-PBA nanocubes (about 500–700 nm, Fig. 2d) on each other [39]. After phosphating, the Ni_2_P/FeP_2_/MN maintained with the spherical Rubik’s cube morphology, but the surface became rough and the corners of the cubes were passivated (Figs. 2e and S14d–f). As contrasts, the morphologies of FeP_2_/MN and Ni_2_P/MN were also investigated. As shown in Fig. S15a–c, the aggregated nanocubes of FeP_2_/MN were unevenly distributed and collapsed, while the morphology of Ni_2_P/MN was the nanoparticles grown on nanosheets (Fig. S15d–f). To verify the heterostructure of Ni_2_P/FeP_2_, analysis of high-resolution transmission electron microscopy (HRTEM) of Ni_2_P/FeP_2_/MN (Fig. 2f) was carried out, in which the fringe spacing of 0.234 nm (101) and 0.515 nm (100) can be ascribed to FeP_2_ and Ni_2_P, respectively. Notably, the decent heterojunction interface in Ni_2_P/FeP_2_ is the origin of constructing IPF. Figure 2g is the transmission electron microscopy (TEM) mapping of target sample, in which Fe, Ni, O and P were homogeneous distributed.

**Fig. 3:**
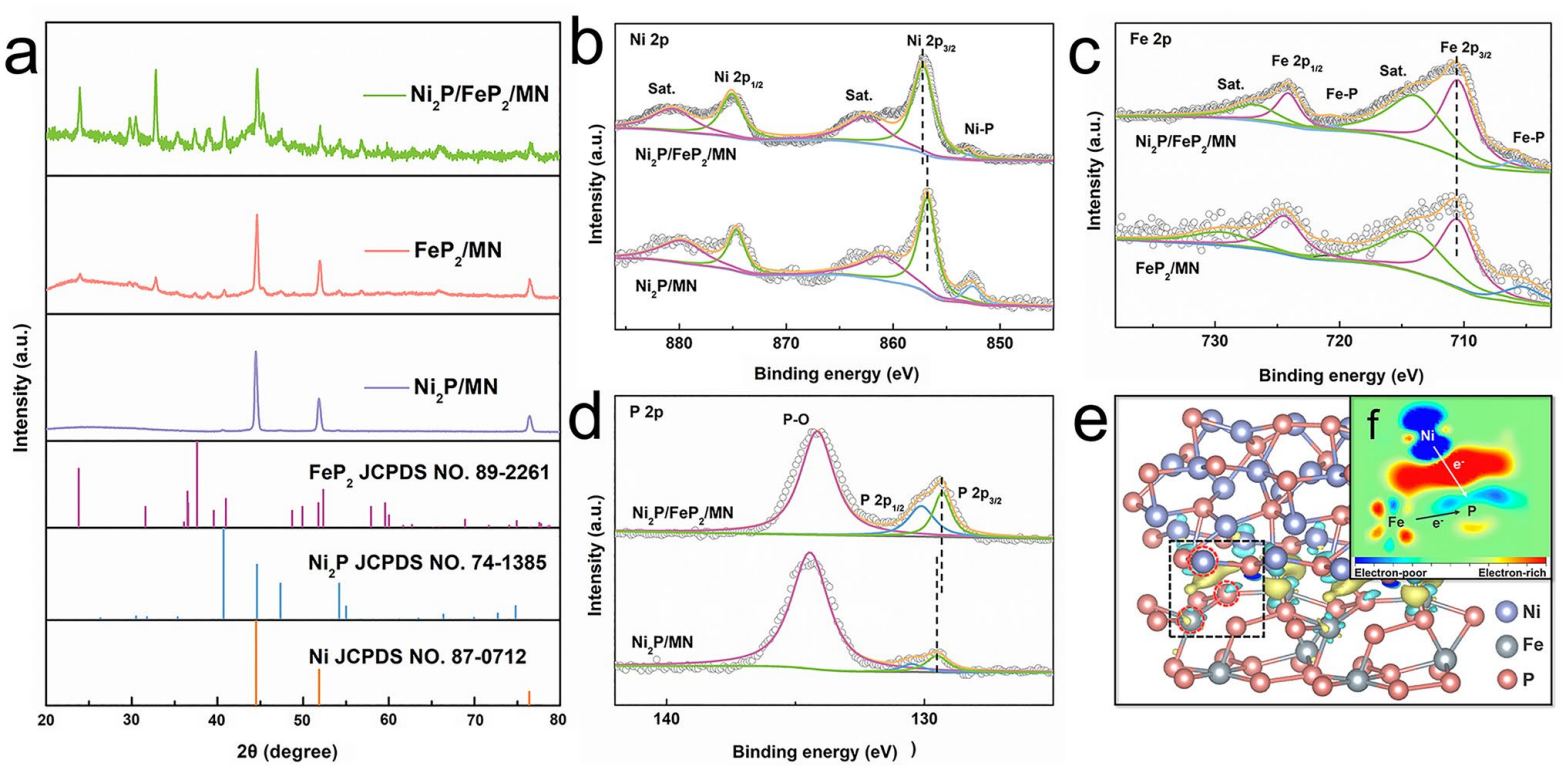
**a** XRD of Ni_2_P/FeP_2_/MN, FeP_2_/MN and Ni_2_P/MN; XPS of Ni_2_P/FeP_2_/MN, FeP_2_/MN and Ni_2_P/MN: **b** Ni 2*p*, **c** Fe 2*p*, **d** P 2*p*; **e** Electron density difference, **f** the extracted 2D data plot for Ni_2_P/FeP_2_ model

Additionally, the crystal structure of the Ni_2_P/FeP_2_/MN and other contrast samples were verified by X-ray diffraction (XRD) in Figs. 3a and S16. The dominating diffraction peaks of Ni_2_P/FeP_2_/MN approximated at 23.8°, 36.5°, 37.6° and 52.3° belongs to the (111), (120), (101) and (211) crystal planes of FeP_2_ (PDF No. 89–2261), while peaks approximated at 40.7°, 44.6°, 47.4° and 54.2° belongs to the (111), (201), (210) and (300) crystal planes of Ni_2_P (PDF No. 74–1385), indicating the successful synthesis of heterostructure of Ni_2_P and FeP_2_, which is consistent with the result of HRTEM. The surface and chemical valence states of Ni_2_P/FeP_2_ were further examined by X-ray photoelectron spectroscopy (XPS). In the high-resolution Ni 2*p* spectrum (Fig. 3b), compared with pure Ni_2_P, an obvious positive shift can be observed in the Ni 2*p*_3/2_ (857.3 eV) and Ni 2*p*_1/2_ (875.1 eV) peaks of Ni_2_P/FeP_2_/MN, resulting in a higher oxidation state of Ni atoms [38]. By contrast, there is no significant shift of Fe 2*p* in Fig. 3c, implying that the construction of heterojunction has weak regulation on the electronic structure of Fe. As depicted in Figs. 3d and S17, lower binding energy peaks located at 129.3 and 130.1 eV are ascribed to P 2*p*_3/2_ and P 2*p*_1/2_ in Ni_2_P/FeP_2_, indicating the bond between P and Ni/Fe, which coincident with the result form XRD [40]. Besides, there is a slightly negative shift compared with that of pure Ni_2_P, indicating that the electron transfer from metal, especially Ni, to phosphorus. As a result, XPS certified that the construction of heterojunction interface can facilitate the redistribution of electron in Ni and P, which may further optimize the adsorption energy of intermediate in OER process. The electron transfer behavior was further investigated by the calculated charge density difference (Figs. 3e and S18), with the electron-poor (blue) and electron-rich (yellow) regions. Coupling Ni_2_P/FeP_2_ led to strong electron interaction at the interface and local electrophilic/nucleophilic region. The extracted 2D data plot (Fig. 3f) displayed the electron accumulation and depletion areas were mainly existed between Ni and P, which is consistent with the result of XPS [41].

### OER Performance in Alkaline Water Electrolyzer (AWE)

**Fig. 4:**
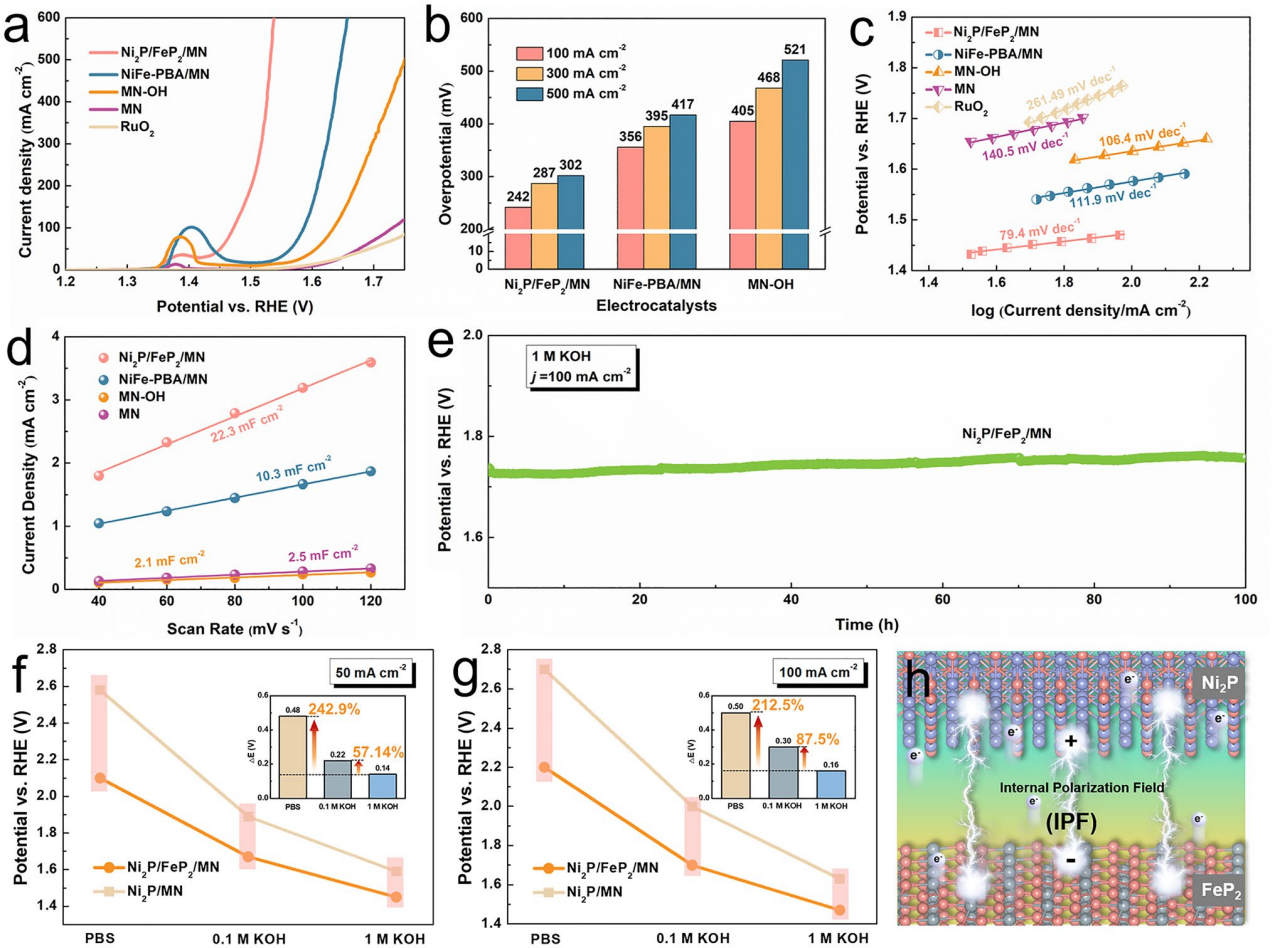
**a** Electrochemical measurements of different catalysts in 1 M KOH. **a** LSV curves; **b** Overpotential comparison of obtained catalysts at 100/300/500 mA cm^−2^; **c** Tafel plots; **d** C_dl_ values; **e** Chronopotentiometric curve of Ni_2_P/FeP_2_/MN obtained at the constant current densities of 100 mA cm ^−2^ in 1 M KOH. Overpotentials of Ni_2_P/FeP_2_/MN and Ni_2_P/MN in PBS, 0.1 M KOH and 1.0 M KOH at **f** 50 mA cm^−2^ and **g** 100 mA cm^−2^ in AWE system (inset: difference values of overpotential in different electrolyte). **h** Formation of a vertical built-in electric field from Ni_2_P to FeP_2_

The OER performances of materials in AWE system were first characterized from linear scan voltammetry (LSV) with corresponding Tafel plots in 1 M KOH electrolyte. Ni_2_P/FeP_2_/MN exhibited superior OER activity and kinetics (Fig. 4a–c) with a low *ƞ*_100_, 100 mA cm^–2^ of 242 mV and Tafel slope of 79.4 mV dec^−1^, as compared with those of NiFe-PBA/MN (356 mV, 111.9 mV dec^−1^) and MN–OH (405 mV, 106.4 mV dec^−1^). As depicted in Fig. S19, the Ni_2_P/FeP_2_/MN material was comparable with the state-of-the-art TM-based electrocatalysts. Besides, the Ni_2_P/FeP_2_/MN catalyst could achieve 500 mA cm^–2^ only requiring the overpotential of 302 mV (Fig. 4b), demonstrating its potential for application in large current density. The reason for the improvement of intrinsic catalytic activity is inferred via contrast samples of FeP_2_/MN and Ni_2_P/MN. The specific activities of Ni_2_P/FeP_2_/MN and other contrast samples are compared in Table S1, which further proves that Ni_2_P/FeP_2_/MN possessed higher intrinsic activity. As illustrated in Fig. S20, the oxidation peak of nickel species in the Ni_2_P/FeP_2_/MN is greatly suppressed compared with that of Ni_2_P/MN, indicating that the construction of heterojunction interface could regulate the electronic structure of Ni atoms and increase the content of high-oxidation-state nickel species, which is consistent with the results of XPS [42–44]. The catalytic activity of catalysts was evaluated by the electrochemical active surface area (ECSA), which can be calculated by the double-layer capacitance (*C*_*d*l_) [45]. As shown in Figs. 4d and S21, cyclic voltammetry (CV) curves and corresponding fitted C_*d*l_ were analyzed. Ni_2_P/FeP_2_/MN (22.3 mF cm^–2^) displays the higher fitted capacitance than those of NiFe-PBA/MN (10.3 mF cm^–2^), MN–OH (2.1 mF cm^–2^) and MN (2.5 mF cm^–2^), suggesting the higher ECSA of Ni_2_P/FeP_2_/MN for OER. Besides, compared with MN, MN–OH with similar C_*d*l_ value possess higher intrinsic activity. Moreover, the potential of Ni_2_P/FeP_2_/MN only decreased 1.67% after 100 h under the current density of 100 mA cm^–2^ (Fig. 4e). After long-term stability test, the Ni_2_P/FeP_2_/MN material still preserved most of the spherical Rubik’s cube structure (Fig. S22), and the XRD of Ni_2_P/FeP_2_/MN after stability test (Fig. S23) maintained most of the dominating peaks. Notably, the P-M signal is significantly weakened and the P-O signal is enhanced, indicating the leaching of P element and intense oxidation process during OER (Fig. S24) [39].

In order to further explore the OH^−^ capture ability of Ni_2_P/FeP_2_/MN with hydroxyl dual-channel, the contrast OER measurements were performed on the heterostructure and Ni_2_P/MN in electrolytes with different concentrate of OH^−^ (PBS, 0.1 M KOH, 1.0 M KOH) in AWE system (Fig. S25). The potentials that the catalysts needed to achieve 50 mA cm^–2^ were illustrated in Fig. 4f, as well as the potential differences (ΔE) in different electrolyte. Experimental results show that the ΔE between Ni_2_P/FeP_2_/MN and Ni_2_P/MN is 0.14 V when the current density reaches 50 mA cm^–2^ in 1.0 M KOH. As the concentrate of OH^−^ decreased, the value of ΔE increased. Compared with Δ*E* in 1.0 M KOH, the ΔE between heterostructure and Ni_2_P/MN in 0.1 M KOH (0.22 V) and PBS (0.48 V) are promoted 57.14 and 242.9%, respectively. Besides, similar regulation can be observed when current density reached 100 mA cm^–2^ (Fig. 4g). It is speculated that these differences are mainly due to the spillover effect of hydroxyl by dual channel under the IPF (Fig. 4h), which is an interesting phenomenon. The experimental data of hydroxyl dual-channel were further analyzed. When the concentration of OH^−^ is high, CN I and CN II synergistically transmit hydroxyl, in which CN I plays a dominant role. On the other hand, when the concentration of OH^−^ is low, CN II plays a significant role. We speculated the origin cause of Δ*E* amplification. The captured OH^−^ via FeP_2_ spontaneously transport to the Ni site in Ni_2_P under the induction of IPF, providing sufficient hydroxyl supply to the nickel active site, widening the activity gap between Ni_2_P/FeP_2_/MN and Ni_2_P/MN.

### OER Performance in Anion Exchange Membrane Water Electrolyzer (AEMWE)

**Fig. 5:**
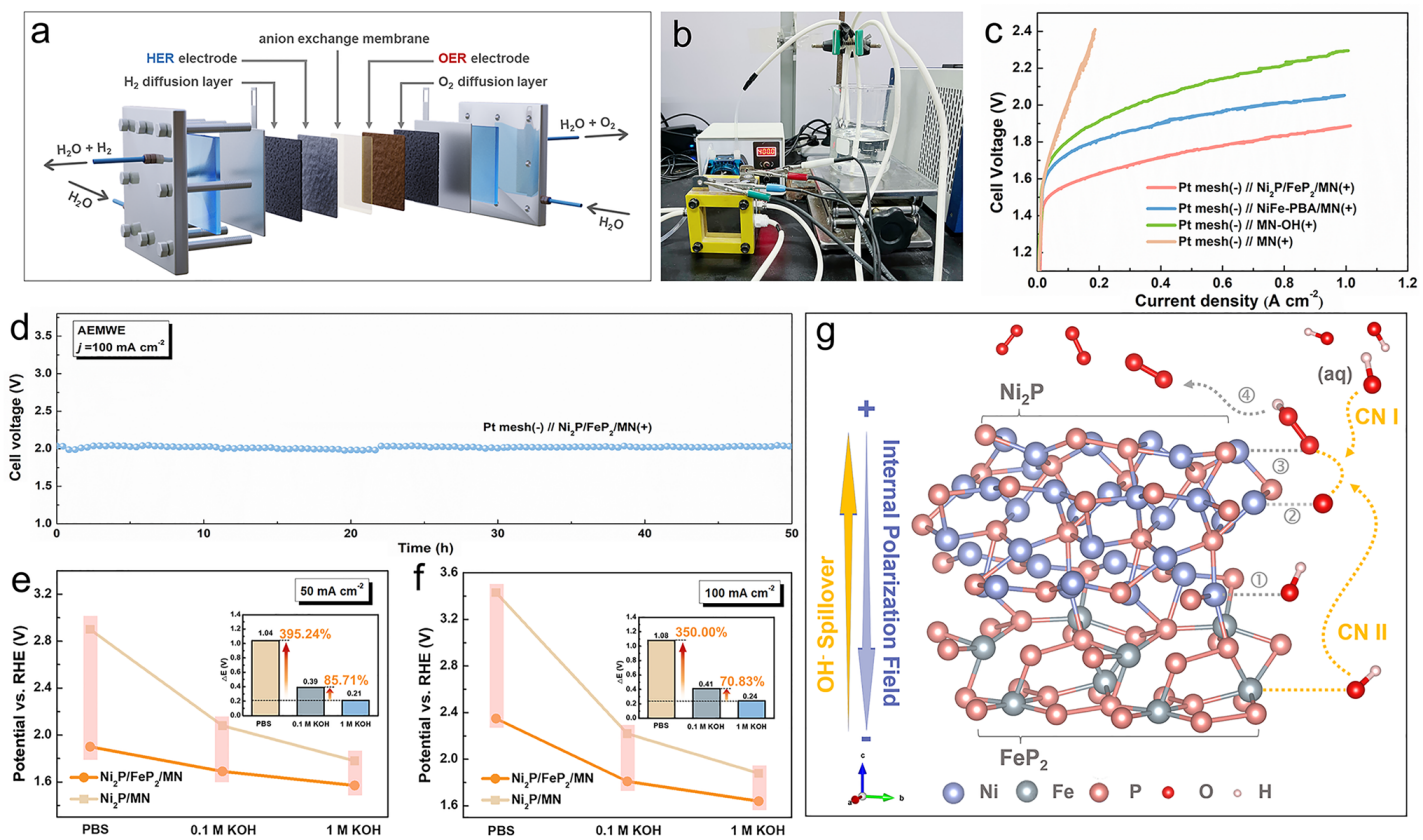
**a** Diagram of anion exchange membrane water electrolyzer (AEMWE); **b** AEMWE device. **c** Polarization curves of AEM water electrolyzer with Ni_2_P/FeP_2_@PA/MN, Ni_2_P/FeP_2_/MN, NiFe-PBA/MN, MN–OH and MN as the anode, the Pt mesh as the cathode in 1 M KOH solution at 25 ℃. **d** Stability of Pt mesh(−) // Ni_2_P/FeP_2_@PA/MN( +) at 100 mA cm^−2^ in AEMWE. Overpotentials of Ni_2_P/FeP_2_/MN and Ni_2_P/MN in PBS, 0.1 M KOH and 1.0 M KOH at **e** 50 mA cm^−2^ and **f** 100 mA cm^−2^ in AEMWE system (inset: difference values of overpotential in different electrolyte). **g** Mechanism diagram of dual channel hydroxyl transport for Ni_2_P/FeP_2_ system based on IPF

The key problem of insufficient OH^−^ supply is more obvious in the AEMWE system due to the sluggish mass transfer process in low concentrated KOH, which greatly limits the overall efficiency of hydrogen production [46]. Therefore, the lab-scale AEMWE single-cell system (Fig. 5a) was designed and built to explore a series of experiment. The AEMWE system device built in the laboratory is shown in the Fig. 5b. The OER performances of materials in AEMWE system were first characterized by polarization curves in 1 M KOH solution at 25 ℃. The synthesized Ni_2_P/FeP_2_/MN, NiFe-PBA/MN and MN–OH were used as OER electrocatalysts on the anode, while Pt mesh was used as the cathode. As illustrated in Fig. 5c, the performances of Ni_2_P/FeP_2_/MN, NiFe-PBA/MN, MN–OH and MN at the AEM electrolyzer level show similar trend as the performance evaluated at the alkaline water electrolyzer level. Ni_2_P/FeP_2_/MN as the anode in AEM electrolyzer delivers the superior activity. In order to reach a practically valuable current density of 1.0 A cm^–2^ in the electrolysis of AEM, the cell voltage of 1.88 V (Ni_2_P/FeP_2_/MN), 2.05 V (NiFe-PBA/MN) and 2.29 V (MN–OH) is needed (Fig. S26), respectively. The voltage of FeP_2_/MN (2.21 V) and Ni_2_P/MN (2.39 V) was also measured (Fig. S27). Besides, Table S2 listed the reported AEM electrocatalysts under large catalytic current densities. The stability of the AEMWE system catalyzed by Ni_2_P/FeP_2_/MN was tested at a current density of 100 mA cm^–2^ for 50 h in Fig. 5d. Similarly, the cell efficiency remained almost constant after the long-term durability test.

In order to further explore the spillover effect of hydroxyl by dual channel under the IPF, a series of experiment was designed in AEMWE system. Ni_2_P/FeP_2_/MN and Ni_2_P/MN were used as the anode in different concentrate of OH^−^ (PBS, 0.1 M KOH, 1.0 M KOH) and the electrodes activation were measured by polarization curves (Fig. S28). The potential differences of the catalysts at the current density of 50 mA cm^–2^ in above electrolytes were illustrated in Fig. 5e. Experimental results show that the Δ*E* between Ni_2_P/FeP_2_/MN and Ni_2_P/MN is 0.21 V in 1.0 M KOH. The value of Δ*E* increases with decreasing OH^−^ concentration. The Δ*E* in 0.1 M KOH (0.39 V) and PBS (1.04 V) are promoted 85.71 and 395.24% when compared with the Δ*E* in 1.0 M KOH. Besides, when the current density reaches 100 mA cm^–2^ (Fig. 5f), the trend of change is similar to that of 50 mA cm^–2^. The ΔE in 0.1 M KOH (0.41 V) and PBS (1.08 V) are still promoted 70.83 and 350.00%, respectively. The comparison of the values of Δ*E* in AWE and AEMWE system are more obvious in Table S3 and S4. Interestingly, compared with AWE system, the ΔE of heterostructure Ni_2_P/FeP_2_/MN in AEMWE system exhibits greater difference from Ni_2_P/MN in the condition of 0.1 M KOH and PBS, which means that the spillover effect of hydroxyl in CN II channel is amplified (Fig. 5g), in which the existence of IPF would be a key factor in ion-transfer-determining process. The interesting phenomenon proves that the HOSo effect from heterojunction structure has better application in low concentration alkaline electrolyte environment. In summary, there are three indicators for designing this type of catalysts: i) *Φ*_acceptor_ < *Φ*_activator_; ii) Δ*G*_OH*(acceptor)_ < 0; iii) Δ*G*_OH*(activator)_ > 0. This finding provides a new idea for the design of catalysts suitable for both high and low OH^−^ concentration electrolytes in AWE and AEMWE systems.

## Conclusion

In summary, we have utilized the HOSo effect from a Ni_2_P/FeP_2_ heterostructure eco-platform to boost OER process. Theoretical profiles make sense the spontaneous hydroxyl adsorption of FeP_2_ and hydroxyl dual-channel transmission, still featuring the excellent catalytic activity for both AWE and AEMWE system limited by hydroxyl supply. Taking advantage by IPF and spontaneous hydroxyl adsorption, the heterostructure can realize the lower* ƞ*_100_ (100 mA cm^–2^) overpotentials of 242 mV, due to the oriented HOSo from FeP_2_ to Ni_2_P which can facilitate the hydroxyl diffusion and provided a new pathway to optimize OOH* formation energy in RDS on Ni active site. Encouragingly, the advantage of HOSo effect is obviously amplified in assembled AEMWE system, especially when the concentration of electrolyte is low. HOSo effect revealed by this research opens new insights for designing highly active non-precious electrocatalysts in different alkaline electrolyte concentrations, promoting the developing of hydrogen economy.

## Supplementary Information

Below is the link to the electronic supplementary material.

Supplementary file1 (PDF 2129 KB)

## References

[1] S. Stiber, H. Balzer, A. Wierhake, F.J. Wirkert, J. Roth et al., Porous transport layers for proton exchange membrane electrolysis under extreme conditions of current density, temperature, and pressure. Adv. Energy Mater. **11**, 2100630 (2021). 10.1002/aenm.202100630

[2] H. Wang, J. Gao, C. Chen, W. Zhao, Z. Zhang et al., PtNi-W/C with atomically dispersed tungsten sites toward boosted ORR in proton exchange membrane fuel cell devices. Nano-Micro Lett. **15**, 143 (2023). 10.1007/s40820-023-01102-9 10.1007/s40820-023-01102-9 PMC10236083 37266746

[3] K.G. Santos, C.T. Eckert, E. Rossi, R.A. Bariccatti, E.P. Frigo et al., Hydrogen production in the electrolysis of water in Brazil, a review. Renew. Sust. Energ. Rev. **68**, 563 (2017). https://www.sciencedirect.com/science/article/pii/S1364032116306372

[4] O. Schmidt, A. Gambhir, I. Staffell, A. Hawkes, J. Nelson et al., Future cost and performance of water electrolysis: an expert elicitation study. Int. J. Hydrogen Energy **42**(52), 30470 (2017). 10.1016/j.ijhydene.2017.10.045

[5] P. Thangavel, M. Ha, S. Kumaraguru, A. Meena, A.N. Singh et al., Graphene-nanoplatelets-supported NiFe-MOF: high-efficiency and ultra-stable oxygen electrodes for sustained alkaline anion exchange membrane water electrolysis. Energy Environ. Sci. **13**(10), 3447 (2020). 10.1039/D0EE00877J

[6] Z.W. Seh, J. Kibsgaard, C.F. Dickens, I. Chorkendorff, J.K. Nørskov et al., Combining theory and experiment in electrocatalysis: insights into materials design. Science **355**(6321), eaad4998 (2017). 10.1126/science.aad4998 10.1126/science.aad4998 28082532

[7] F. Song, L. Bai, A. Moysiadou, S. Lee, C. Hu et al., Transition metal oxides as electrocatalysts for the oxygen evolution reaction in alkaline solutions: an application-inspired renaissance. J. Am. Chem. Soc. **140**(25), 7748 (2018). 10.1021/jacs.8b04546 29788720 10.1021/jacs.8b04546

[8] B. Guo, Y. Ding, H. Huo, X. Wen, X. Ren et al., Recent advances of transition metal basic salts for electrocatalytic oxygen evolution reaction and overall water electrolysis. Nano-Micro Lett. **15**, 57 (2023). 10.1007/s40820-023-01038-0 10.1007/s40820-023-01038-0 PMC9981861 36862225

[9] Y.P. Zhu, T.Y. Ma, M. Jaroniec, S.Z. Qiao et al., Self-templating synthesis of hollow Co_3_O_4_ microtube arrays for highly efficient water electrolysis. Angew. Chem. Int. Ed. **56**(5), 1324 (2017). 10.1002/anie.201610413 10.1002/anie.201610413 27900829

[10] J. Li, J. Li, J. Ren, H. Hong, D. Liu et al., Electric-field-treated Ni/Co_3_O_4_ film as high-performance bifunctional electrocatalysts for efficient overall water splitting. Nano-Micro Lett. **14**, 148 (2022). 10.1007/s40820-022-00889-3 10.1007/s40820-022-00889-3 PMC9307702 35869313

[11] Q. Zhou, C. Xu, J. Hou, W. Ma, T. Jian et al., Duplex interpenetrating-phase FeNiZn and FeNi_3_ heterostructure with low-Gibbs free energy interface coupling for highly efficient overall water splitting. Nano-Micro Lett. **15**, 95 (2023). 10.1007/s40820-023-01066-w 10.1007/s40820-023-01066-w PMC10086094 37037951

[12] A. Lončar, D. Escalera-López, S. Cherevko, N. Hodnik, Inter-relationships between oxygen evolution and Iridium dissolution mechanisms. Angew. Chem. Int. Ed. **61**(14), e202114437 (2022). 10.1002/anie.202114437 10.1002/anie.202114437 PMC9305877 34942052

[13] C. Wang, Q. Zhang, B. Yan, B. You, J. Zheng et al., Facet engineering of advanced electrocatalysts toward hydrogen/oxygen evolution reactions. Nano-Micro Lett. **15**, 52 (2023). 10.1007/s40820-023-01024-6 10.1007/s40820-023-01024-6 PMC9935811 36795218

[14] J.J. Song, C. Wei, Z.F. Huang, C.T. Liu, X. Wang et al., A review on fundamentals for designing oxygen evolution electrocatalysts. Chem. Soc. Rev. **49**(7), 2196 (2020). 10.1039/C9CS00607A 32133479 10.1039/c9cs00607a

[15] Z.F. Huang, J. Song, Y. Du, S. Xi, S. Dou et al., Chemical and structural origin of lattice oxygen oxidation in Co–Zn oxyhydroxide oxygen evolution electrocatalysts. Nat. Energy **4**(4), 329 (2019). 10.1038/s41560-019-0355-9

[16] J.T. Li, Oxygen evolution reaction in energy conversion and storage: design strategies under and beyond the energy scaling relationship. Nano-Micro Lett. **14**(1), 112 (2022). 10.1007/s40820-022-00857-x 10.1007/s40820-022-00857-x PMC9051012 35482112

[17] I. Vincent, A. Kruger, D. Bessarabov, Development of efficient membrane electrode assembly for low cost hydrogen production by anion exchange membrane electrolysis. Int. J. Hydrogen Energy **42**(16), 10752 (2017). https://www.sciencedirect.com/science/article/pii/S036031991730993X

[18] J. Hnát, M. Plevová, J. Žitka, M. Paidar, K. Bouzek, Anion-selective materials with 1,4-diazabicyclo[2.2.2]octane functional groups for advanced alkaline water electrolysis. electrochim. Acta **248**, 547 (2017). https://www.sciencedirect.com/science/article/pii/S0013468617316031

[19] T.T. Wang, X. Li, Y.J. Pang, X.R. Gao, Z.K. Kou et al., Unlocking the synergy of interface and oxygen vacancy by core-shell nickel phosphide@oxyhydroxide nanosheets arrays for accelerating alkaline oxygen evolution kinetics. Chem. Eng. J. **425**, 131491 (2021). https://www.sciencedirect.com/science/article/pii/S1385894721030722

[20] C. Hu, L. Dai, Multifunctional carbon-based metal-free electrocatalysts for simultaneous oxygen reduction, oxygen evolution, and hydrogen evolution. Adv. Mater. **29**(9), 1604942 (2017). 10.1002/adma.201604942 10.1002/adma.201604942 28009458

[21] N.U. Hassan, M. Mandal, G. Huang, H.A. Firouzjaie, P.A. Kohl, Achieving high-performance and 2000 h stability in anion exchange membrane fuel cells by manipulating ionomer properties and electrode optimization. Adv. Energy Mater. **10**(40), 2001986 (2020). 10.1002/aenm.202001986

[22] A. Kumar, V.Q. Bui, J. Lee, A.R. Jadhav, Y. Hwang et al., Modulating interfacial charge density of NiP_2_–FeP_2_ via coupling with metallic Cu for accelerating alkaline hydrogen evolution. ACS Energy Lett. **6**(2), 354 (2021). 10.1021/acsenergylett.0c02498

[23] D. Li, A.R. Motz, C. Bae, C. Fujimoto, G. Yang et al., Durability of anion exchange membrane water electrolyzers. Energy Environ. Sci. **14**(6), 3393 (2021). 10.1039/D0EE04086J

[24] I.V. Pushkareva, A.S. Pushkarev, S.A. Grigoriev, P. Modisha, D.G. Bessarabov, Comparative study of anion exchange membranes for low-cost water electrolysis. Int. J. Hydrogen Energy **45**(49), 26070 (2020). https://www.sciencedirect.com/science/article/pii/S0360319919341588

[25] O. Heijden, S. Park, J. Eggebeen, M. Koper, Non-kinetic effects convolute activity and tafel analysis for the alkaline oxygen evolution reaction on NiFeOOH electrocatalysts. Angew. Chem. Int. Ed. **62**(7), e202216477 (2022). 10.1002/anie.202216477 10.1002/anie.202216477 PMC10108042 36533712

[26] S. Lee, K. Banjac, M. Lingenfelder, X. Hu, Oxygen isotope labeling experiments reveal different reaction sites for the oxygen evolution reaction on nickel and nickel iron oxides. Angew. Chem. Int. Ed. **58**(30), 10295 (2019). 10.1002/anie.201903200 10.1002/anie.201903200 PMC6771717 31106463

[27] L. An, J. Feng, Y. Zhang, R. Wang, H. Liu et al., Epitaxial heterogeneous interfaces on N-NiMoO_4_/NiS_2_ nanowires/nanosheets to boost hydrogen and oxygen production for overall water splitting. Adv. Funct. Mater. **29**(1), 1805298 (2019). 10.1002/adfm.201805298

[28] P. Phonsuksawang, P. Khajondetchairit, T. Butburee, S. Sattayaporn, N. Chanlek et al., Effects of Fe doping on enhancing electrochemical properties of NiCo_2_S_4_ supercapacitor electrode. Electrochim. Acta **340**, 135939 (2020). https://www.sciencedirect.com/science/article/pii/S0013468620303315

[29] D. Liang, C. Lian, Q. Xu, M. Liu, H. Liu et al., Interfacial charge polarization in Co_2_P_2_O_7_@N, P Co-doped carbon nanocages as Mott-Schottky electrocatalysts for accelerating oxygen evolution reaction. Appl. Catal. B **268**, 118417 (2020). https://www.sciencedirect.com/science/article/pii/S0926337319311634

[30] Y. Liu, Y. Chen, Y. Tian, T. Sakthivel, H. Liu et al., Synergizing hydrogen spillover and deprotonation by the internal polarization field in a MoS_2_/NiPS_3_ vertical heterostructure for boosted water electrolysis. Adv. Mater. **34**(37), 2203615 (2022). 10.1002/adma.202203615 10.1002/adma.202203615 35900215

[31] C. Lyu, J. Cheng, K. Wu, J. Wu, N. Wang, Interfacial electronic structure modulation of CoP nanowires with FeP nanosheets for enhanced hydrogen evolution under alkaline water/seawater electrolytes. Appl. Catal. B **317**, 121799 (2022). https://www.sciencedirect.com/science/article/pii/S0926337322007408

[32] X. Wang, X. Zong, B. Liu, G. Long, A. Wang et al., Boosting electrochemical water oxidation on NiFe (oxy) hydroxides by constructing schottky junction toward water electrolysis under industrial conditions. Small **18**(4), 2105544 (2022). 10.1002/smll.202105544 10.1002/smll.202105544 34841659

[33] W.J. Sun, H.Q. Ji, L.X. Li, H.Y. Zhang, Z.K. Wang et al., Built-in electric field triggered interfacial accumulation effect for efficient nitrate removal at ultra-low concentration and electroreduction to ammonia. Angew. Chem. Int. Ed. **60**(42), 22933 (2021). 10.1002/anie.202109785 10.1002/anie.202109785 34431192

[34] Y. Kim, M. Ha, R. Anand, M. Zafari, J.M. Baik et al., Unveiling a surface electronic descriptor for Fe–Co mixing enhanced the stability and efficiency of perovskite oxygen evolution electrocatalysts. ACS Catal. **12**(23), 14698 (2022). 10.1021/acscatal.2c04424

[35] Q. Wen, K. Yang, D. Huang, G. Cheng, X. Ai et al., Schottky heterojunction nanosheet array achieving high-current-density oxygen evolution for industrial water splitting electrolyzers. Adv. Energy Mater. **11**(46), 2102353 (2021). 10.1002/aenm.202102353

[36] A. Zagalskaya, V. Alexandrov, Role of defects in the interplay between adsorbate evolving and lattice oxygen mechanisms of the oxygen evolution reaction in RuO_2_ and IrO_2_. ACS Catal. **10**(6), 3650 (2020). 10.1021/acscatal.9b05544

[37] Y. Lin, Z. Liu, L. Yu, G.R. Zhang, H. Tan et al., Overall oxygen electrocatalysis on nitrogen-modified carbon catalysts: identification of active sites and in situ observation of reactive intermediates. Angew. Chem. Int. Ed. **60**(6), 3299 (2021). 10.1002/anie.202012615 10.1002/anie.202012615 PMC7898341 33151593

[38] P. Wang, R. Qin, P. Ji, Z. Pu, J. Zhu et al., Synergistic coupling of Ni nanoparticles with Ni_3_C nanosheets for highly efficient overall water splitting. Small **16**(37), 2001642 (2020). 10.1002/smll.202001642 10.1002/smll.202001642 32762000

[39] X. Luo, P. Ji, P. Wang, X. Tan, L. Chen et al., Spherical Ni_3_S_2_/Fe–NiP_x_ magic cube with ultrahigh water/seawater oxidation efficiency. Adv. Sci. **9**(7), 2104846 (2022). 10.1002/advs.202104846 10.1002/advs.202104846 PMC8895145 35243823

[40] T. Wu, S. Zhang, K. Bu, W. Zhao, Q. Bi et al., Nickel nitride–black phosphorus heterostructure nanosheets for boosting the electrocatalytic activity toward the oxygen evolution reaction. J. Mater. Chem. A **7**(38), 22063 (2019). 10.1039/C9TA07962A

[41] Y. Liu, J. Zhang, Y. Li, Q. Qian, Z. Li et al., Realizing the synergy of interface engineering and chemical substitution for Ni_3_N enables its bifunctionality toward hydrazine oxidation assisted energy-saving hydrogen production. Adv. Funct. Mater. **31**(35), 2103673 (2021). 10.1002/adfm.202103673

[42] C. Kuai, C. Xi, A. Hu, Y. Zhang, Z. Xu et al., Revealing the dynamics and roles of iron incorporation in nickel hydroxide water oxidation catalysts. J. Am. Chem. Soc. **143**(44), 18519 (2021). 10.1021/jacs.1c07975 34641670 10.1021/jacs.1c07975

[43] L. Trotochaud, S.L. Young, J.K. Ranney, S.W. Boettcher, Nickel–iron oxyhydroxide oxygen-evolution electrocatalysts: the role of intentional and incidental iron incorporation. J. Am. Chem. Soc. **136**(18), 6744 (2014). 10.1021/ja502379c 24779732 10.1021/ja502379c

[44] Y. Bai, Y. Wu, X. Zhou, Y. Ye, K. Nie et al., Promoting nickel oxidation state transitions in single-layer NiFeB hydroxide nanosheets for efficient oxygen evolution. Nat. Commun. **13**(1), 6094 (2022). 10.1038/s41467-022-33846-0 36241751 10.1038/s41467-022-33846-0 PMC9568589

[45] Y. Li, C.K. Peng, H. Hu, S.Y. Chen, J.H. Choi et al., Interstitial boron-triggered electron-deficient Os aerogels for enhanced pH-universal hydrogen evolution. Nat. Commun. **13**(1), 1143 (2022). 10.1038/s41467-022-28805-8 35241652 10.1038/s41467-022-28805-8 PMC8894469

[46] Y. Yang, P. Li, X. Zheng, W. Sun, S.X. Dou et al., Anion-exchange membrane water electrolyzers and fuel cells. Chem. Soc. Rev. **51**(23), 9620 (2022). 10.1039/D2CS00038E 36345857 10.1039/d2cs00038e

