# Supplementary material for: Internal Polarization Field Induced Hydroxyl Spillover Effect for Industrial Water Splitting Electrolyzers
Source: Nanomicro Lett. 2023 Nov 30;16:39. doi: 10.1007/s40820-023-01253-9 (PMC10689691; doi:10.1007/s40820-023-01253-9)
Supplement: Supplementary file 1 — Supplementary file1 (PDF 2129 KB) [file 40820_2023_1253_MOESM1_ESM.pdf]

Supporting Information for

Internal Polarization Field Induced Hydroxyl Spillover Effect for Industrial Water Splitting Electrolyzers

Jingyi Xie[1], Fuli Wang[1], Yanan Zhou[1], Yiwen Dong[1], Yongming Chai[1,]* and Bin Dong[1,]*

[1] State Key Laboratory of Heavy Oil Processing, College of Chemistry and Chemical Engineering, China University of Petroleum (East China), Qingdao 266580, P. R. China

*Corresponding author. E-mail: ymchai@upc.edu.cn (Yongming Chai); dongbin@upc.edu.cn (Bin Dong)

S1 Experimental Procedures

S1.1 Materials Preparation

Chemicals: molybdenum nickel foam (MN, Thick of 1.5 mm), Sodium chloride (NaCl). Potassium ferricyanide ($K_3[Fe(CN)_6]$, ≥99%), Sodium hypophosphite monohydrate ($NaH_2PO_2 \cdot H_2O$, ≥99%), Phytic acid ($C_6H_{18}O_{24}P_6$), Potassium hydroxide (KOH) were purchased from Sinopharm Chemical Reagent Co. Ltd.. MN was cut into pieces of 1 × 1.5 cm^2 and then wished with hydrochloric acid, acetone, absolute ethyl alcohol and deionized water, respectively for later use.

Preparation of MN-OH and NiFe-PBA/MN nanocubes: In a typical process, MN-OH was obtained by immersing a piece of molybdenum nickel foam in 50 mM NaCl solution at room temperature for 12 h. Then, MN-OH was placed in a Teflon-lined stainless steel autoclave containing 0.6 mmol $K_3[Fe(CN)_6]$ and 30 mL deionized water. After heating for several hours at 90 °C, the product was named as NiFe-PBA/MN-t (t is the hydrothermal time) and rinsed with deionized water.

Preparation of Ni_2P/FeP_2/MN nanocubes: The dried NiFe-PBA/MN-t and $NaH_2PO_2 \cdot H_2O$ were placed in a tube furnace and calcined under argon atmosphere for 2 h at 350 °C. The product was obtained and denoted as Ni_2P/FeP_2/MN-t.

S1.2 Materials Characterization

Structural and morphological characterization: To characterize the chemical structures, Fourier transform infrared (FTIR) spectra were collected on a Bruker V70FTIR spectrometer. X-ray diffraction (XRD) patterns were obtained on a JSM-7500F X-ray diffractometer. X-ray photoelectron spectroscopy (XPS) were carried out on a VG ESCALABMK II scanning X-ray spectroscope. In order to reduce the influence of Ni element on the substrate, the surface layer of the catalyst growing on the substrate was scraped off for XPS testing. Energy dispersive X-ray (EDX)

mapping images were characterized using JEOL JEM-2100F field emission electron microscope operating at 200 kV. Transmission electron microscopy (TEM) was acquired on a FEI Tecnai G_2 F20 S-TWIN. Scanning electron microscopy (SEM) spectra were obtained with a Hitachi (S-4800) cold field emission scanning electron microscope.

S1.3 Electrochemical Measurements

Electrochemical measurements: An electrochemical workstation (Gamry Reference 3000) was used to evolute the electrochemical properties of as-prepared catalysts in 1.0 M and 6.0 M KOH at room temperature. Oxygen is fed into the electrolyte for 30 minutes to get O_2 saturated alkaline condition. The obtained samples, a Pt plate and a Hg/HgO electrode were used as the working electrode, counter electrode and reference electrode, respectively. All the mentioned potentials were converted into reversible hydrogen electrode (RHE) according to the Nernst equation: $E_{RHE} = E_{Hg/HgO} + 0.0594\ pH + 0.095$. The pH of 1 M and 6 M KOH is around 14 and 14.8, respectively. Linear sweep voltammetry (LSV) curves were obtained with a scan rate of 5 $mV{\cdot}s^{-1}$. The Tafel slopes were calculated according to the LSV method. The electrical double-layer capacitances (C_{dl}) was calculated by cyclic voltammetry (CV) curves with different scan rates of 40, 60, 80, 100, 120 mV s^{-1}. The stability was assessed by chronopotentiometry at 100 and 500 mA cm^{-2}.

Alkaline AEM electrolyzer: Firstly, Fumasep FAA-3-50 membrane (130 μm thickness) was activated in 1.0 M KOH for 24 h, and then preserved in distilled water for later use. As-prepared catalyts were used as an anode electrode, and Pt mesh was used as cathode electrode. The whole system was operated at the temperature of 25 ℃ using 1.0 M KOH electrolyte under the flowing rate of 400 $mL{\cdot}min^{-1}$. The circulation of electrolyte was carried out by water pump (kamoer, DIPump550). Besides, the performance was evaluated by measuring polarization curves from 1 to 2.4 V vs. RHE. The stability was evaluated by measuring chronopotentiometry at 100 mA cm^{-2} for 50 h.

S2 Computational Methods

Density functional theory (DFT) computational: The computational calculation was performed within the framework of the DFT as implemented in the Vienna Ab initio Software Package (VASP 5.4.4) code within the Perdew–Burke–Ernzerhof (PBE) generalized gradient approximation and the projected augmented wave (PAW) method [S1−S3]. The cutoff energy for the plane-wave basis set was set to 450 eV. The convergence criterion for the electronic self-consistent iteration and force was set to 10^{-5} eV and 0.02 eV $Å^{-1}$, respectively. Using the Monkhorst-Pack special k-point meshes to carry out Brillouin zone sampling [S4]. 4 × 2 × 1, 5 × 2 × 1, and 5 × 2 × 2 k-point grids were used for Ni_2P, FeP_2, and Ni_2P/FeP_2 system, respectively. A vacuum region of 15 Å was used to avoid the interaction with the upper structure. The atomic structures and charge density differences were visualized via the VESTA code. The absorption free energy intermediate of (ΔG) was calculated according to the following

formulas:

$$\Delta G = \Delta E_{ads} + \Delta E_{ZPE} - T\Delta S$$

where the ΔE_{ads} is the adsorption energy that obtained from DFT calculations, ΔE_{ZPE} is the zero-point energy difference between the adsorbed state of the system and gas phase state, and ΔS is the entropy difference.

The transition state (TS) searches on the catalysts were carried out with the climbing-image nudged elastic band method (CI-NEB) [S5].

S3 Supplementary Figures and Tables

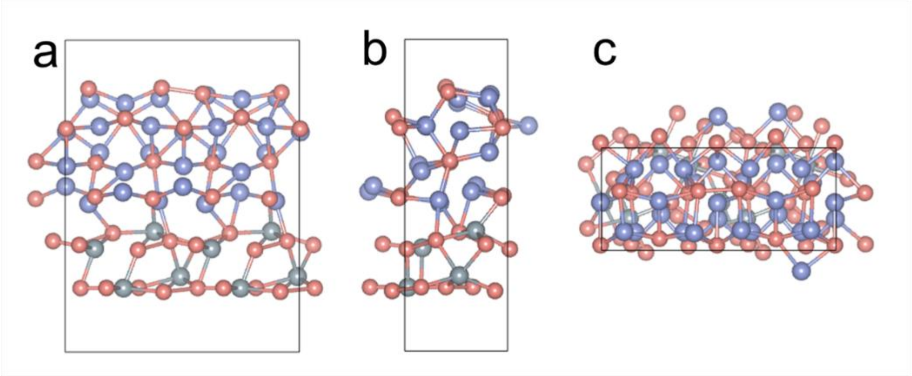

Fig. S1 DFT simulations of Ni_2P/FeP_2 heterogeneous structure configurations from different views: (**a**) front view; (**b**) side view; (**c**) Top view

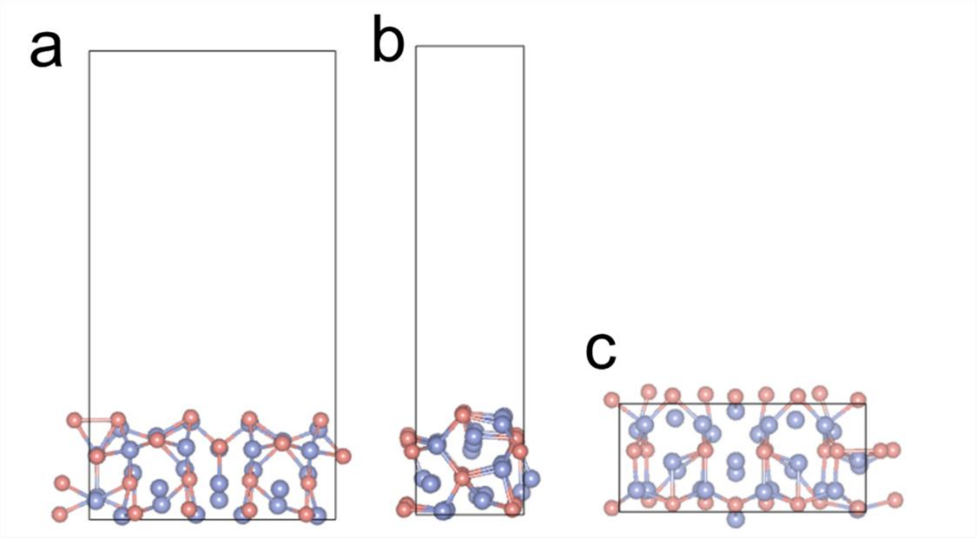

Fig. S2 DFT simulations of Ni_2P heterogeneous structure configurations from different views: (**a**) front view; (**b**) side view; (**c**) Top view

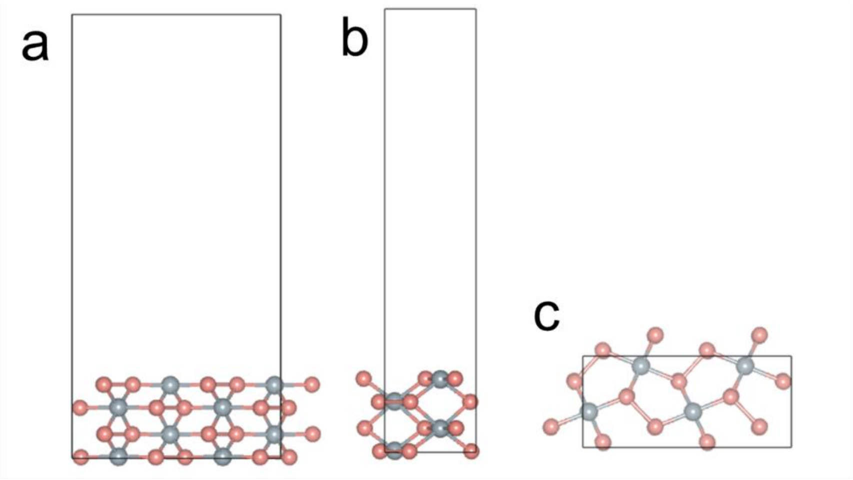

Fig. S3 DFT simulations of FeP_2 structure configurations from different views: (**a**) front view; (**b**) side view; (**c**) Top view

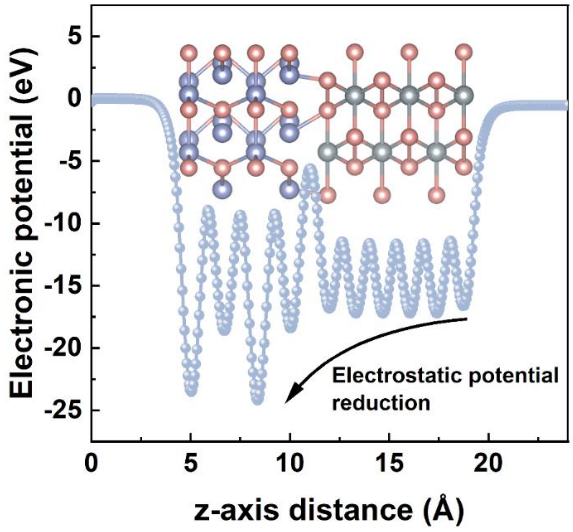

Fig. S4 Planar average potential along the Z-direction of Ni_2P/FeP_2

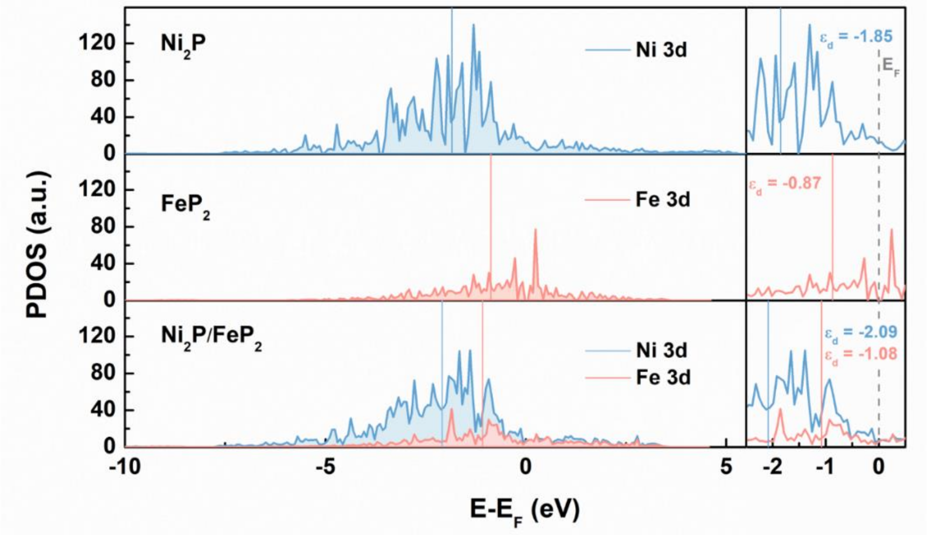

Fig. S5 PDOSs of FeP_2, Ni_2P and Ni_2P/FeP_2

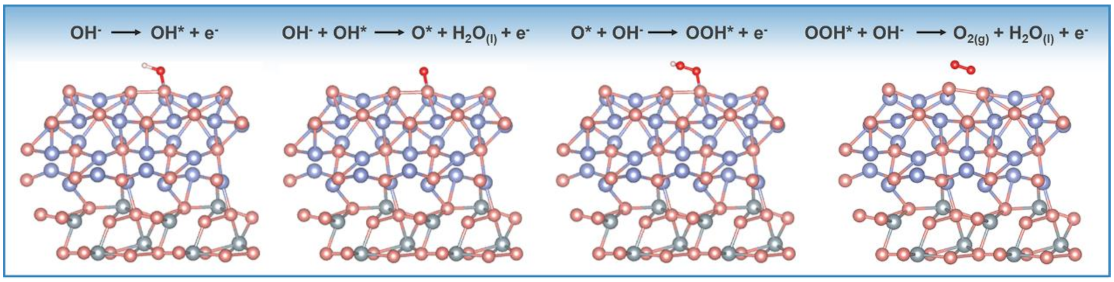

Fig. S6 Proposed four-step OER mechanism for Ni_2P/FeP_2 with Ni active site (act_{Ni})

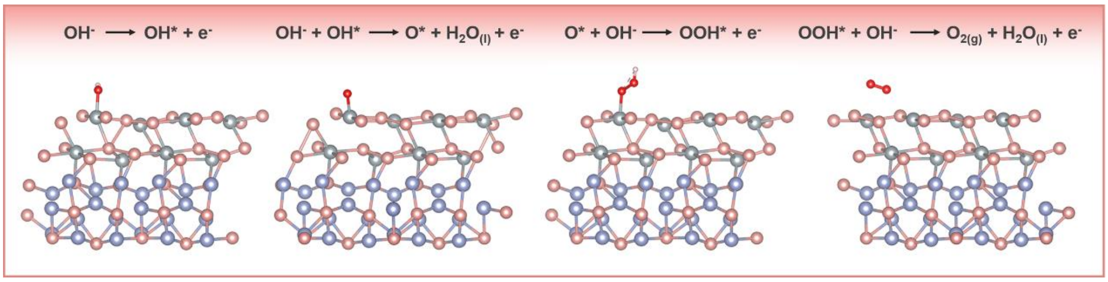

Fig. S7 Proposed four-step OER mechanism for Ni_2P/FeP_2 with Fe active site (act_{Fe})

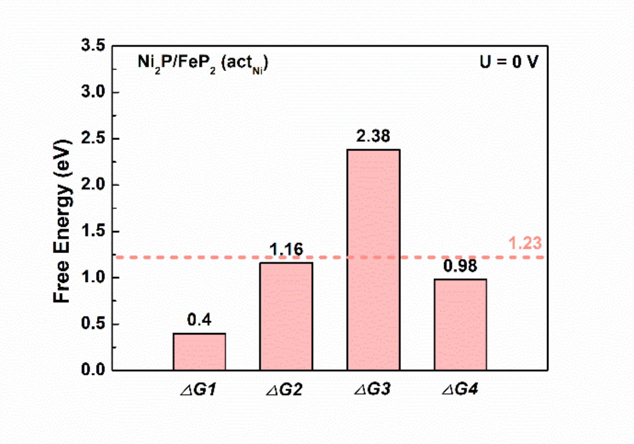

Fig. S8 Gibbs free energies of Ni_2P/FeP_2 with Ni active site (act_{Ni})

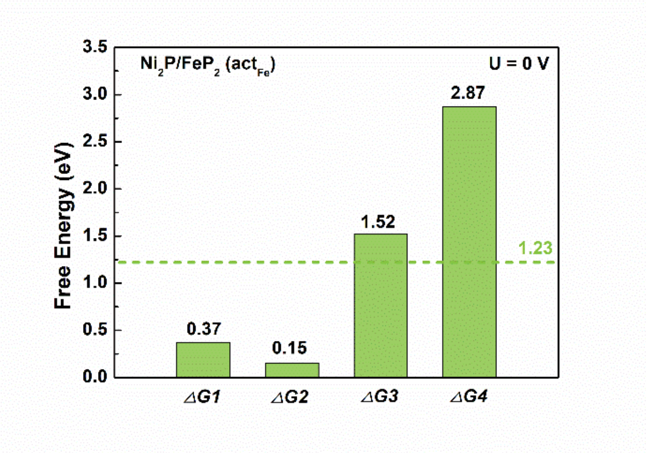

Fig. S9 Gibbs free energies of Ni_2P/FeP_2 with Fe active site (act_{Fe})

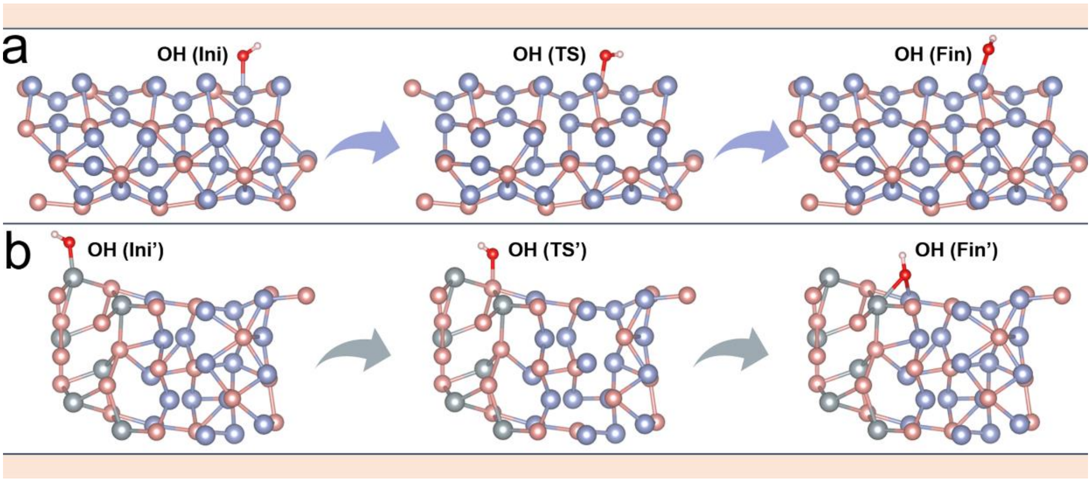

Fig. S10 Interfacial hydroxyl spillover routes of (a) Ni_2P and (b) Ni_2P/FeP_2

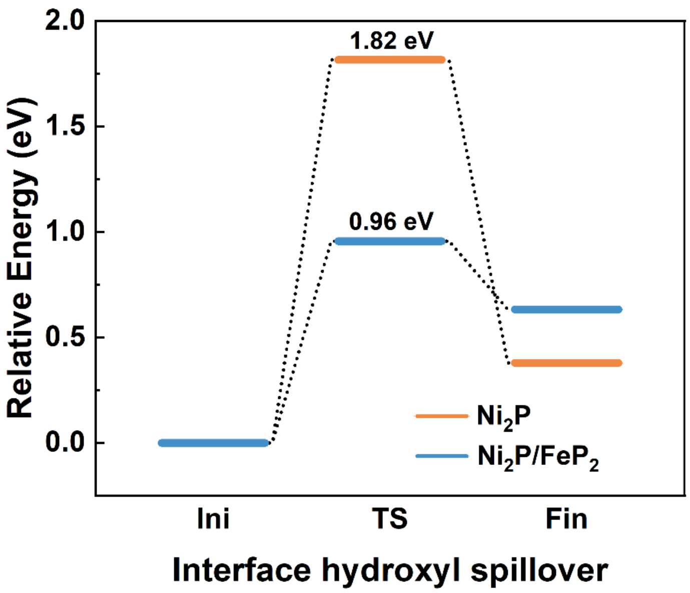

Fig. S11 Overflow energy barrier of Ni_2P/FeP_2 and Ni_2P

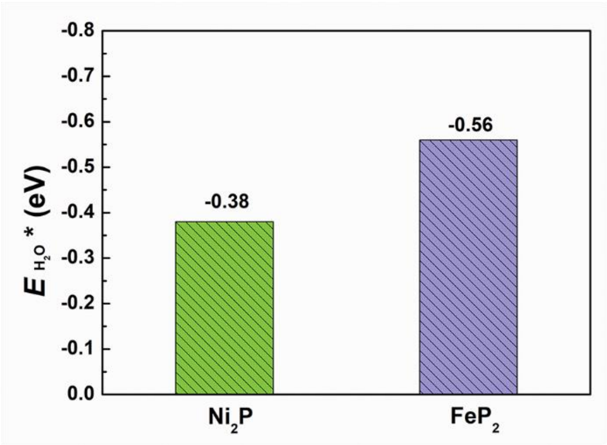

Fig. S12 The adsorption energies of H_2O on the surface of Ni_2P and FeP_2

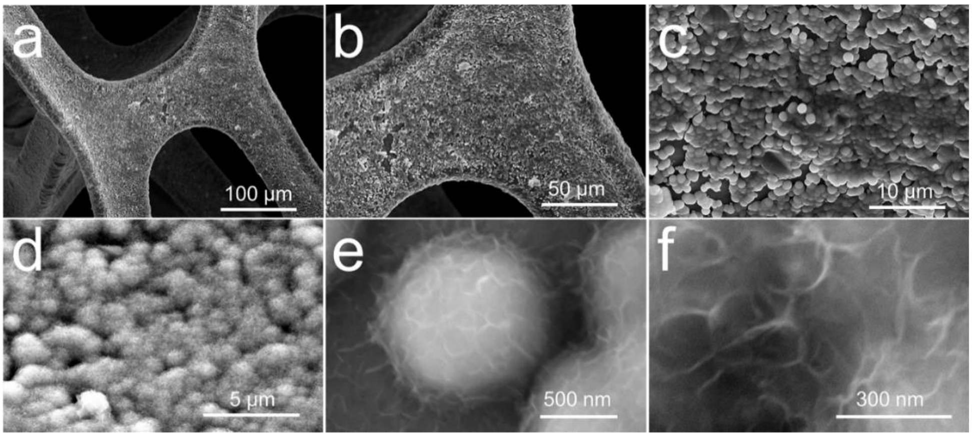

Fig. S13 SEM images of (**a-c**) MN, (**d-f**) MN-OH

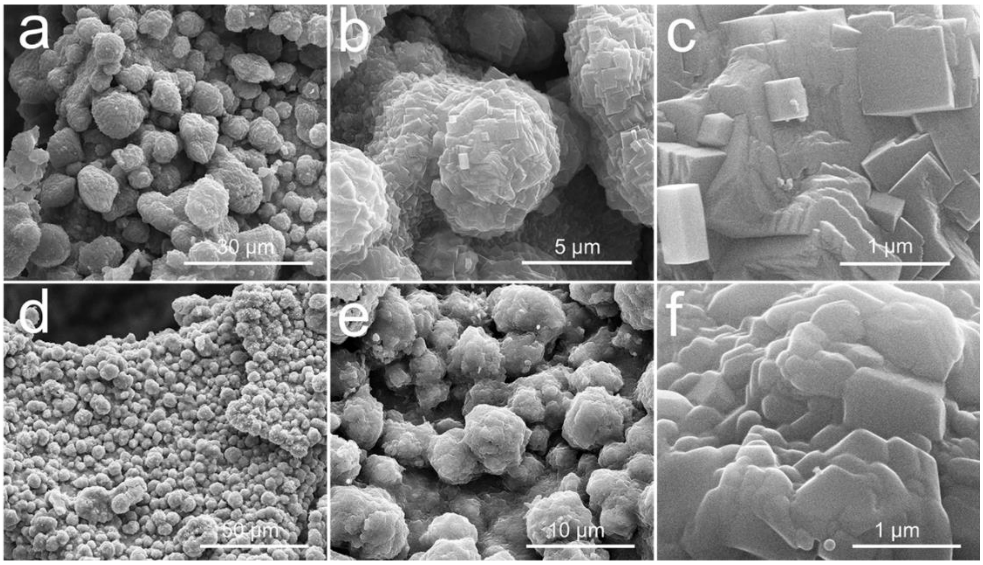

Fig. S14 SEM images of (**a-c**) NiFe-PBA/MN, (**d-f**) Ni_2P/FeP_2/MN

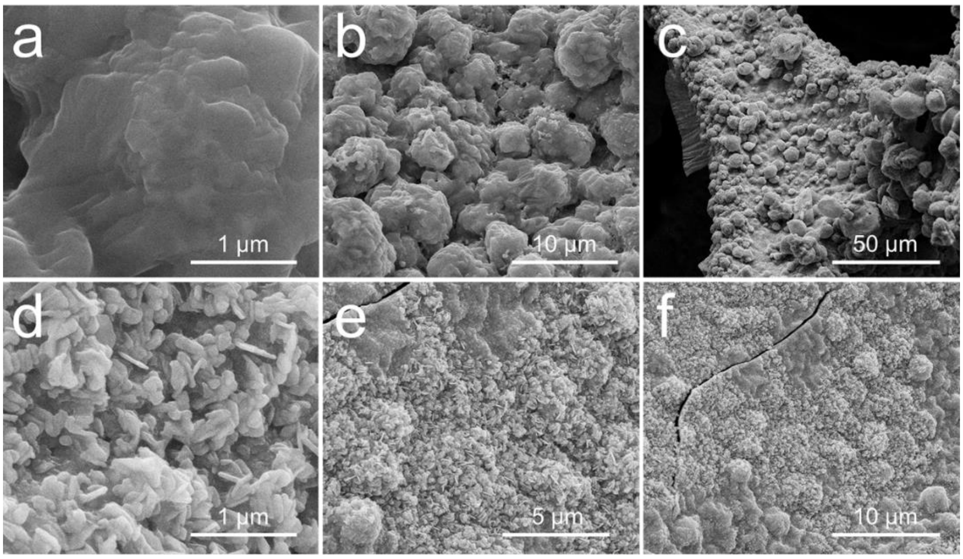

Fig. S15 SEM images of (a-c) FeP_2/MN and (d-f) Ni_2P/MN

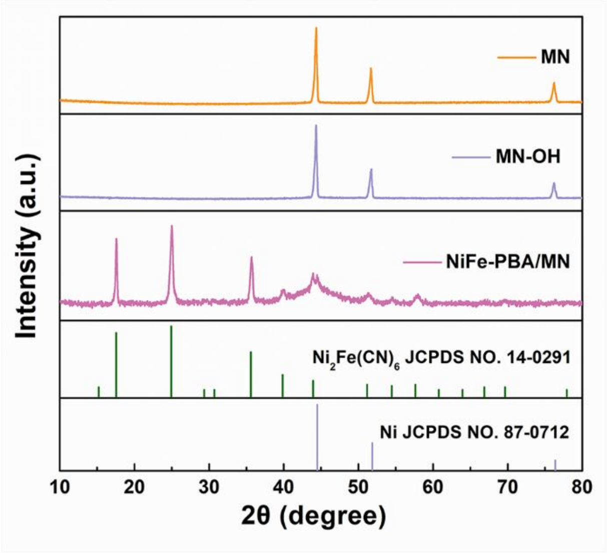

Fig. S16 XRD of MN, MN-OH, NiFe-PBA/MN

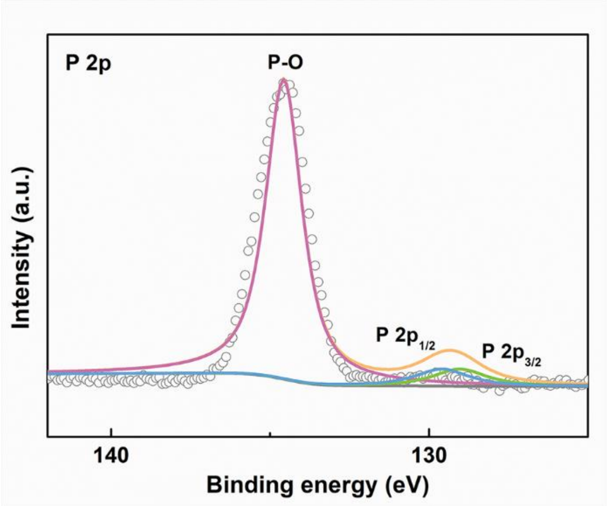

Fig. S17 XPS of P of FeP_2/MN

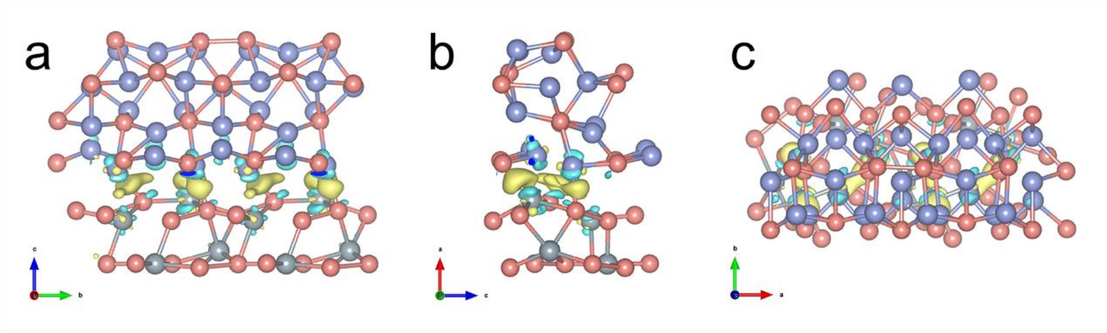

Fig. S18 Electron density difference from different views: (**a**) front view; (**b**) side view; (**c**) Top view

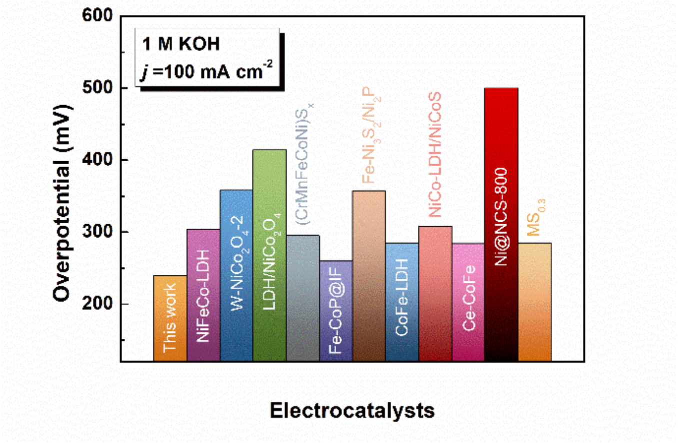

Fig. S19 Overpotential comparison of Ni_2P/FeP_2@PA/MN and catalysts in other works [S6–S16]

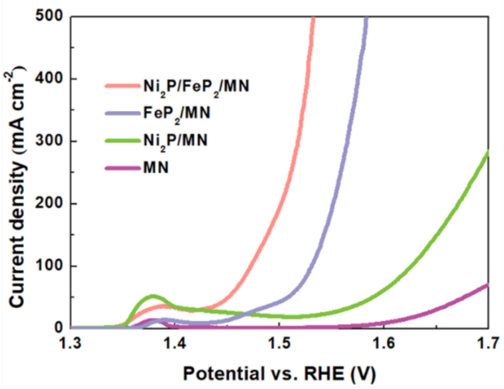

Fig. S20 LSV curves of Ni_2P/FeP_2/MN, FeP_2/MN, Ni_2P/MN and MN in 1.0 M KOH

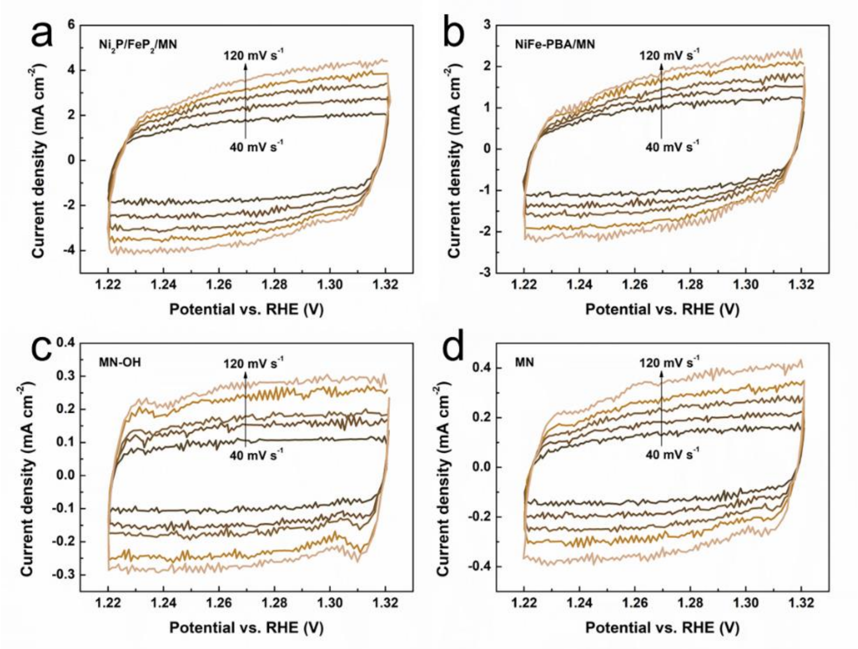

Fig. S21 Cyclic voltammetry (CV) curves of Ni_2P/FeP_2/MN, NiFe-PBA/MN, MN-OH and MN

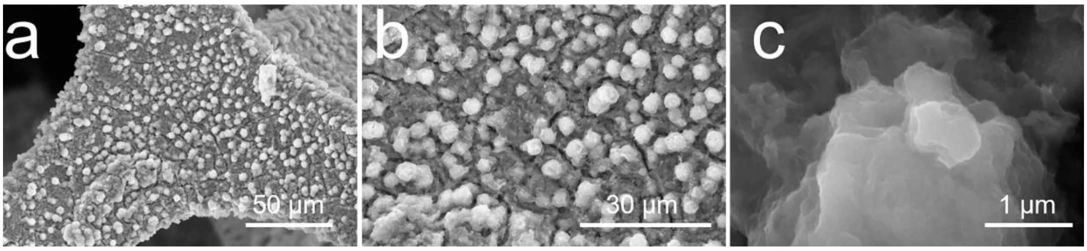

Fig. S22 SEM images of Ni_2P/FeP_2/MN after stability test

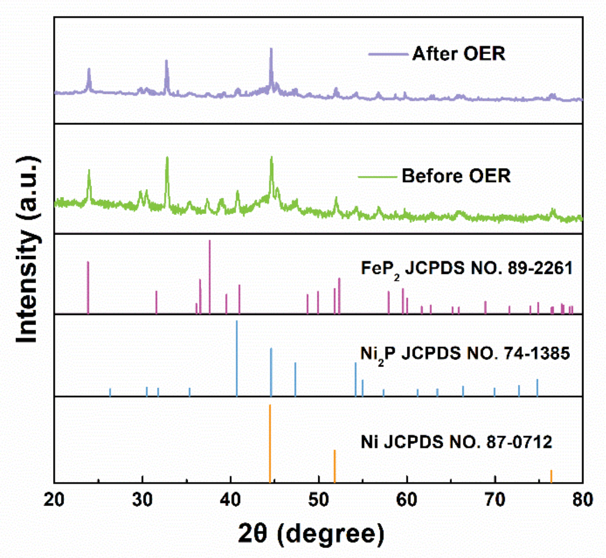

Fig. S23 XRD of NiFe-PBA/MN before and after OER

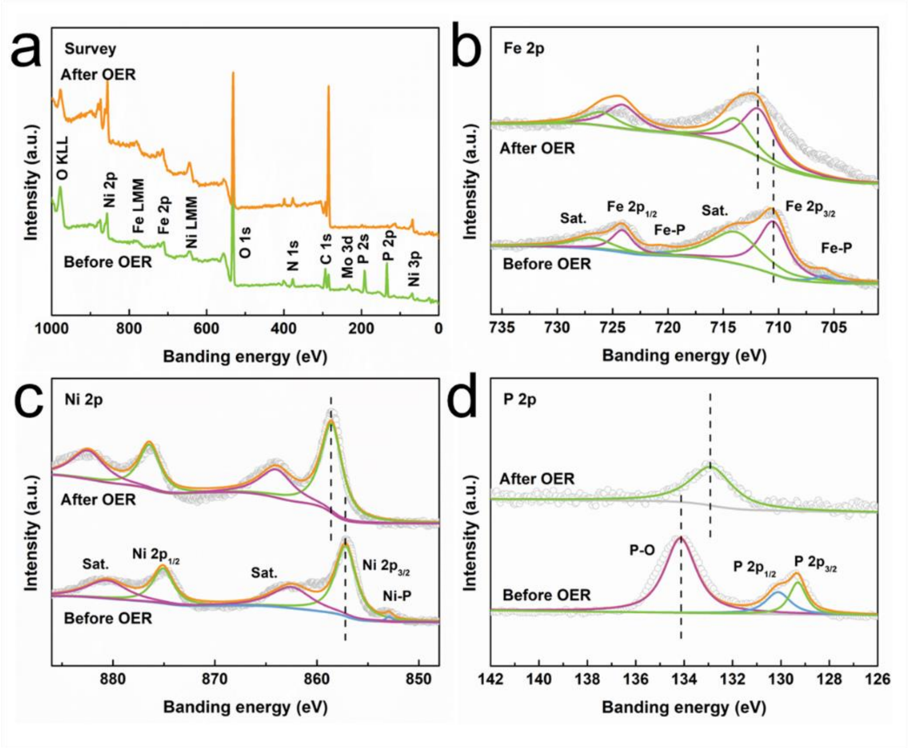

Fig. S24 XPS of (**a**) survey, (**b**) Fe, (**c**) Ni, (**d**) P of Ni_2P/FeP_2/MN before and after OER

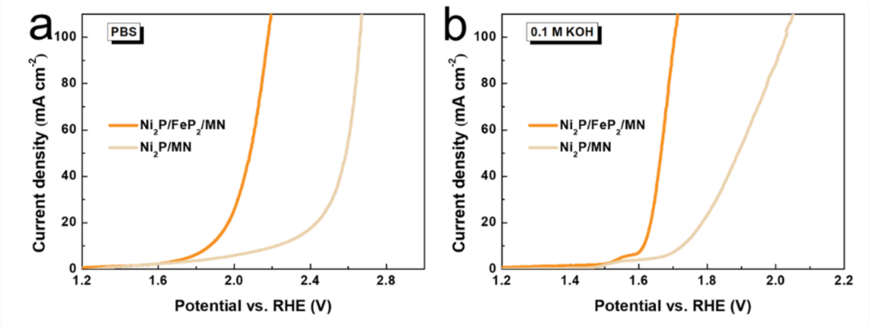

Fig. S25 LSV of $Ni_2P/FeP_2/MN$ and Ni_2P/MN in (a) PBS and (b) 0.1 M KOH in AWE

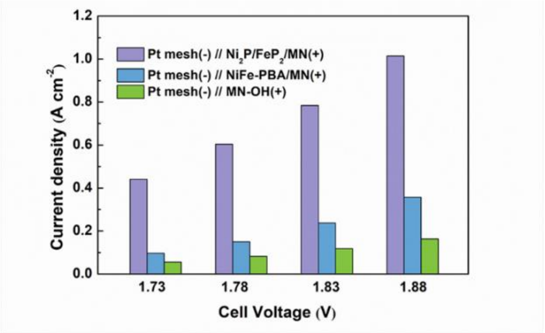

Fig. S26 Current density comparison of obtained catalysts at 1.73/1.78/1.83/1.88 V

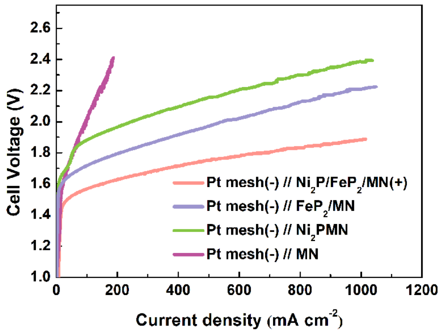

Fig. S27 Polarization curves of $Ni_2P/FeP_2/MN$, FeP_2/MN, Ni_2P/MN and MN (anode), Pt mesh (cathode) in 1.0 M KOH in AEMWE

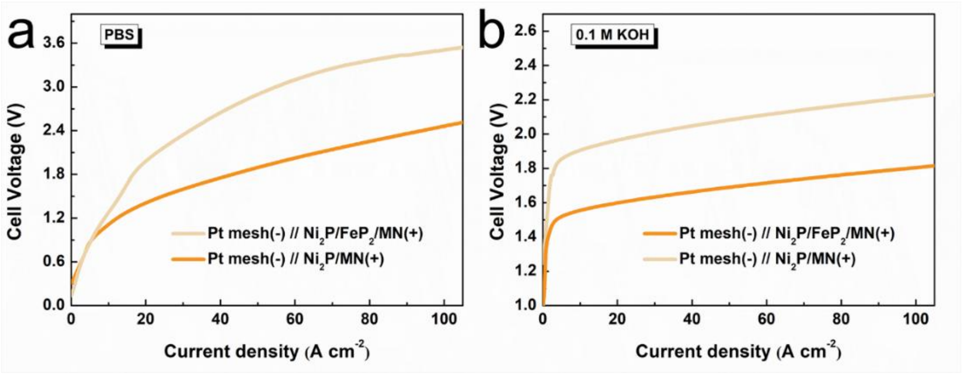

Fig. S28 LSV of $Ni_2P/FeP_2/MN$ and Ni_2P/MN in (**a**) PBS and (**b**) 0.1 M KOH in AEMWE

Table S1 The specific activities of Ni_2P/FeP_2/MN and other catalysts

**Electrocatalysts**	**Specific activity (mA $cm^{-2}{}_{ECSA}$)**
Ni_2P/FeP_2/MN	0.34
NiFe-PBA/MN	0.08
MN-OH	0.18
MN	0.02

Table S2 The comparison of cell voltage of Ni_2P/FeP_2/MN and other catalysts in AEMWE

**Electrocatalysts**	**Cell voltage (V)**		**References**
	**@500 mA cm^{-2}**	**@1000 mA cm^{-2}**	
Ni_2P/FeP_2/MN	1.75	1.88	This work
HS-RuCo/NC	/	2.07	[S17]
NiFeCr-LDH	2.21(200)	/	[S18]
PR-NiFe-LDH	2.13(250)	/	[S19]
d-(Fe,Ni)OOH||NiMoN	1.795	/	[S20]
CuNi@NiSe	/	2.2	[S21]
Ni–Fe CLs	/	2.2(670)	[S22]
$NiCoFeO_x$	2.29	/	[S23]
NiCoO-NCO/C	1.85(504)	/	[S24]
$Ni_2P/Ni_{12}P_5$ (V–$Ni_2P/Ni_{12}P_5$)	1.79	/	[S25]
B, V-Ni_2P	1.78	1.92	[S26]
PdNiFeCo/C-Ceria-$NF_{0.3}$	2.31(300)	/	[S27]

Table S3 The percentage of increased potential (ΔE) of Ni_2P/FeP_2 compared with Ni_2P in AWE and AEMWE system at 50 mA cm^{-2} in different concentrate of OH- electrolyte when compared with 1.0 M KOH solution

Electrolyte	AWE	AEMWE
0.1 M KOH	57.14%	85.71%
PBS	242.9%	395.24%

Table S4 The percentage of increased potential (ΔE) of Ni_2P/FeP_2 compared with Ni_2P in AWE and AEMWE system at 100 mA cm^{-2} in different concentrate of OH- electrolyte when compared with 1.0 M KOH solution

Electrolyte	AWE	AEMWE
0.1 M KOH	87.5%	70.83%
PBS	212.5%	350.00%

Supplementary References

[S1] B. Hammer, L.B. Hansen, J.K. Nørskov, Improved adsorption energetics within density-functional theory using revised perdew-burke-ernzerhof functionals. Phys. Rev. B **59**(11), 7413 (2018). https://link.aps.org/doi/10.1103/PhysRevB.59.7413

[S2] P.E. Blöchl, Projector augmented-wave method. Phys. Rev. B **50**(24), 17953 (1994). https://link.aps.org/doi/10.1103/PhysRevB.50.17953

[S3] G. Kresse, D. Joubert, From ultrasoft pseudopotentials to the projector augmented-wave method. Phys. Rev. B **59**(3), 1758 (1999). https://link.aps.org/doi/10.1103/PhysRevB.59.1758

[S4] H.J. Monkhorst, J.D. Pack, Special points for brillouin-zone integrations. Phys. Rev. B **13**(12), 5188 (1976). https://link.aps.org/doi/10.1103/PhysRevB.13.5188

[S5] G. Henkelman, B.P. Uberuaga, H. Jónsson, A climbing image nudged elastic band method for finding saddle points and minimum energy paths, J. Chem. Phys., **113**, 9901-9904 (2000). https://doi.org/10.1063/1.1329672

[S6] Y.S. Park, J. Jeong, M.J. Jang, C. Kwon, G.H. Kim et al., Ternary layered double hydroxide oxygen evolution reaction electrocatalyst for anion exchange membrane alkaline seawater electrolysis. J. Energy Chem. **75**, 127-134 (2022). https://doi.org/10.1016/j.jechem.2022.08.011

[S7] J, Luo, X, Wang, Y, Gu, S, Wang, Y, Li et al., Hierarchical sheet-like W-doped $NiCo_2O_4$ spinel synthesized by high-valence oxyanion exchange strategy for highly efficient electrocatalytic oxygen evolution reaction. Chem. Eng. J. **472**,

144839 (2023). https://doi.org/10.1016/j.cej.2023.144839

[S8] L. Yang, L. Chen, D. Yang, X. Yu, H. Xue et al., NiMn layered double hydroxide nanosheets/$NiCo_2O_4$ nanowires with surface rich high valence state metal oxide as an efficient electrocatalyst for oxygen evolution reaction. J. Power Sources **392**, 23-32 (2018). https://doi.org/10.1016/j.jpowsour.2018.04.090

[S9] M. Cui, C. Yang, B. Li, Q. Dong, M. Wu et al., High-entropy metal sulfide nanoparticles promise high-performance oxygen evolution reaction. Adv. Energy Mater. **11**, 2002887 (2021). https://doi.org/10.1002/aenm.202002887

[S10] M. Li, Y. Ma, B. Xiao, Y. Zhou, W. Yu et al., S, Fe dual doped and precisely regulated CoP porous nanoneedle arrays for efficient hydrogen evolution at 3 A cm^{-2}. Chem. Eng. J. **470**, 144081 (2023). https://doi.org/10.1016/j.cej.2023.144081

[S11] X. Wang, X. Yu, J. Bai, G. Yuan, P. He et al., Interface engineering assisted Fe-Ni_3S_2/Ni_2P heterostructure as a high-performance bifunctional electrocatalyst for OER and HER. Electrochim Acta **458**, 142524 (2023). https://doi.org/10.1016/j.electacta.2023.142524

[S12] B. Deng, J. Liang, L. Yue, T. Li, Q. Liu et al., CoFe-LDH nanowire arrays on graphite felt: A high-performance oxygen evolution electrocatalyst in alkaline media. Chinese Chem. Lett. **33**, 890-892 (2022). https://doi.org/10.1016/j.cclet.2021.10.002

[S13] J. Li, L. Wang, H. He, Y. Chen, Z. Gao et al., Interface construction of NiCo-LDH/NiCoS based on the 2D ultrathin nanosheet towards oxygen evolution reaction. Nano Res. **15**, 4986-4995 (2022). https://doi.org/10.1007/s12274-022-4144-6

[S14] L. Wang, Y. Liu, X. Liu, W. Chen, 3D nanostructured Ce-doped CoFe-LDH/NF self-supported catalyst for high-performance OER. Dalton Trans. **52**, 12038-12048 (2023). https://doi.org/10.1039/D3DT01814H

[S15] K.B. Patel, B. Parmar, K. Ravi, R. Patidar, J.C. Chaudhari et al., Metal-organic framework derived core-shell nanoparticles as high performance bifunctional electrocatalysts for HER and OER, Appl. Surf. Sci. **616**, 156499 (2023). https://doi.org/10.1016/j.apsusc.2023.156499

[S16] D. Wang, C. Duan, Y. Yu, X. Li, Z. Wang et al., Co-regulation of anion-cation in transition metal high entropy oxide for outstanding OER electrocatalytic performance. J. Alloy. Compd. **967**, 171758 (2023). https://doi.org/10.1016/j.jallcom.2023.171758

[S17] J. Du, D. Chen, Y. Ding, L. Wang, F. Li et al., Highly stable and efficient oxygen evolution electrocatalyst based on Co oxides decorated with ultrafine Ru nanoclusters. Small **19**, 2207611 (2023).

https://doi.org/10.1002/smll.202207611

[S18] M.H. Wang, Z.X. Lou, X. Wu, Y. Liu, J.Y. Zhao et al., Operando high-valence Cr-modified NiFe hydroxides for water oxidation. Small **18**, 2200303 (2022). https://doi.org/10.1002/smll.202200303

[S19] Q. Wen, S. Wang, R. Wang, D. Huang, J. Fang et al., Nanopore-rich NiFe LDH targets the formation of the high-valent nickel for enhanced oxygen evolution reaction. Nano Res. **16**, 2286-2293 (2023). https://doi.org/10.1007/s12274-022-5163-z

[S20] L. Wu, M. Ning, X. Xing, Y. Wang, F. Zhang et al., Boosting oxygen evolution reaction of (Fe,Ni)OOH via defect engineering for anion exchange membrane water electrolysis under industrial conditions. Adv. Mater. 2306097 (2023). https://doi.org/10.1002/adma.202306097

[S21] D. Cao, J. Shao, Y. Cui, L. Zhang, D. Cheng et al., Interfacial engineering of copper-nickel selenide nanodendrites for enhanced overall water splitting in alkali condition. Small **19**, 2301613 (2023). https://doi.org/10.1002/smll.202301613

[S22] E. Fernández, C. Sacedón, J. Rostra, J.P. Espinós, J. Brey et al., Optimization of anion exchange membrane water electrolyzers using ionomer-free electrodes. Renew. Energ. **197**, 1183-1191 (2022). https://doi.org/10.1016/j.renene.2022.08.013

[S23] D. Xu, M.B. Stevens, M.R. Cosby, S.Z. Oener, A.M. Smith et al., Earth-abundant oxygen electrocatalysts for alkaline anion-exchange-membrane water electrolysis: effects of catalyst conductivity and comparison with performance in three-electrode cells. **9**, 7-15 (2019). https://doi.org/10.1021/acscatal.8b04001

[S24] Y.S. Park, J. Jeong, Y. Noh, M.J. Jang, J. Lee et al., Commercial anion exchange membrane water electrolyzer stack through non-precious metal electrocatalysts. Appl. Catal. B-Environ. **292**, 120170 (2021). https://doi.org/10.1016/j.apcatb.2021.120170

[S25] T. Zhao, S. Wang, Y. Li, C. Jia, Z. Su, Heterostructured V-doped $Ni_2P/Ni_{12}P_5$ electrocatalysts for hydrogen evolution in anion exchange membrane water electrolyzers. **18**, 2204758 (2022). https://doi.org/10.1002/smll.202204758

[S26] T. Zhao, S. Wang, C. Jia, C. Rong, Z. Su et al., Cooperative boron and vanadium doping of nickel phosphides for hydrogen evolution in alkaline and anion exchange membrane water/seawater electrolyzers. Small **19**, 2208076 (2023). https://doi.org/10.1002/smll.202208076

[S27] M. Zhiani, F. Jalili, S. Kamali, In situ cathode polarization measurement in alkaline anion exchange membrane water electrolyzer equipped with a PdNiFeCo/C-Ceria hydrogen evolution electrocatalyst. Int. J. Hydrogen Energ. **42**, 26563-26574 (2017). https://doi.org/10.1016/j.ijhydene.2017.09.038
